# Supplementary material for: High-throughput in vivo mapping of RNA accessible interfaces to identify functional sRNA binding sites
Source: Nat Commun. 2018 Oct 4;9:4084. doi: 10.1038/s41467-018-06207-z (PMC6172242; doi:10.1038/s41467-018-06207-z)
Supplement: Supplementary file 1 — Supplementary Information [file 41467_2018_6207_MOESM1_ESM.pdf]

Supplementary Information

High throughput *in vivo* mapping of RNA accessible interfaces to identify functional sRNA binding sites

Mihailovic, et al.

Supplementary Methods

Total RNA extraction

Total RNA was extracted from a sample of 1-5 mL of culture. Briefly, 1 mL of room temperature Trizol (Invitrogen) was used to resuspend each cell pellet upon which samples were left at room temperature for 5 min. Next, 200 µL of 24:1 chloroform: isoamyl alcohol were added to each sample and samples were inverted vigorously for 15 seconds prior to centrifugation at 13,000 xg for 10 min at 4ºC. The aqueous phase was then transferred to 2mL DNase/RNase-free microcentrifuge tubes. Equal volumes of 0.8 M sodium citrate and 1.2 M sodium chloride (~ 300 µL) were added to each sample and inverted for 1 min prior to overnight precipitation at -20ºC. The following day, samples were centrifuged at 13,000 xg at 4ºC for 30 min. The liquid was removed carefully as to not disturb the formed RNA pellet. RNA pellets were then washed twice with 75% ethanol (10 min 13,000 xg centrifugations at 4ºC). After the second wash, liquid was completely removed from the RNA pellet and sample was left to dry in a laminar flow hood for 10 min. Once dry, RNA pellets were resuspended in 30 µL nuclease-free water and RNA concentration was measured via spectrophotometry.

Total RNA samples (diluted to 200 ng/µL) were treated with RNAse-free DNase I (PI-90083 Thermo-Fisher Scientific) following manufacturer protocol. After DNase I treatment, 10 µL of GlycoBlue (AM9516 Life Technologies) was added to an equivalent volume solution of 100% isopropanol and DNase-treated RNA (total 55 µL) and precipitated at -20ºC overnight. The following day, samples were pelleted (15 min at 13,000 xg) and liquid was removed carefully. Next, samples were washed with chilled 95% ethanol. After the second wash, liquid was completely removed from the RNA pellet and sample was left to dry in a laminar flow hood for 10 min. Once dry, RNA pellets were resuspended in 50 µL nuclease-free water and stored at -80ºC. Finally, the quality of RNA was evaluated by using a bioanalyzer (Agilent) at the Genomic Sequencing and Analysis Facility (GSAF at UT Austin) to confirm that no significant degradation had occurred.

PCR Amplification and in vitro Transcription (IVT) for EMSA

Primers were designed to amplify sRNAs and mRNAs (Supplementary Table 5) (with an upstream T7 promoter) from previously-designed plasmids or genomic DNA (*E. coli* BW25113). Specifically, 4 random nucleotides (GACT) were added upstream of the T7 promoter sequence (TAATACGACTCACTATAGGGAGA). PCR product sizes were verified on a 1.5 % agarose gel prior to PCR clean up (GE Illustra GFX PCR).

mRNAs were *in vitro* transcribed using the MEGA Script T7 kit following the manufacturer instructions. Specifically 1-1.5 pmol of DNA template was used per reaction, and each reaction was incubated for 6 hours. Following DNase digestion, one of two reliable RNA recovery methods was performed. If an RNA Clean and Concentrator-5 kit (Zymo Research) was used, 30 µL of nuclease-free water was added to the IVT reaction prior to following manufacturer instruction manual. In the case that RNA was recovered using a chloroform-based method, 115 µL of nuclease-free water and 15 115 µL of Ammonium Acetate Stop Solution (from MEGA Script T7 kit) were added and mixed. Next, two volumes of 25:24:1 phenol: chloroform: isoamyl alcohol were added to each sample and samples were inverted for one minute, then centrifuged at 13,000 xg at 4ºC for 5 min. The top aqueous phase was transferred to two equivalent volumes of 24:1 chloroform: isoamyl alcohol and samples were centrifuged at 13,000 xg at 4ºC for 5 min. The top phase was again transferred, this time to microcentrifuge tubes containing 1 mL isopropyl alcohol and 1.5 µL GlycoBlue each, and left to precipitate overnight at -20ºC.

The following day, samples were centrifuged at 13,000 xg at 4ºC for 15 min. The liquid was removed carefully as to not disturb the formed RNA pellet. One mL of 95% ethanol was added to each sample and the samples were centrifuged at 13,000 xg at 4ºC for 5 min. The liquid was again removed carefully as to not disturb the RNA pellet. One mL of 75% ethanol was added to each sample and the samples were centrifuged for at 13,000 xg at 4ºC for 5 min. The liquid was again removed and samples left to dry in a solvent hood for 2 hours. Once dry, 30 µL of nuclease-free water was added to re-suspend the RNA. RNA concentration was measured via spectrophotometry. To validate transcripts (length and lack of spurious transcripts), 3 µL of each RNA was mixed with 7 µL of nuclease-free water and 5 µL of RNA Loading Buffer II (NEB) and denatured for 5 min at 70ºC. Samples were loaded into an 8% urea gel (SequaGel, National Diagnostics) and run at 100 V for 3 hours. The gel was then stained using Sybr Green II (Thermo-Fisher Scientific) for 30 min and imaged under Sybr Green setting in a gel imager (ChemiDoc XRS +, BioRad).

sRNAs were prepared as were mRNAs with slight modifications to support internal P-32 labeling. Specifically, 0.5-1.5 µL UTP [α-32P] (3000Ci/mmol 10mCi/ml, 250 µCi, PerkinElmer) was added to replace unlabeled UTP from the MEGA Script IVT kit. RNA samples were re-suspended in 20 µL of nuclease-free water and, if RNA was recovered using the chloroform-based method, free NTPs removed using DTR Gel Filtration Cartridges (EdgeBio) following manufacturer instructions.

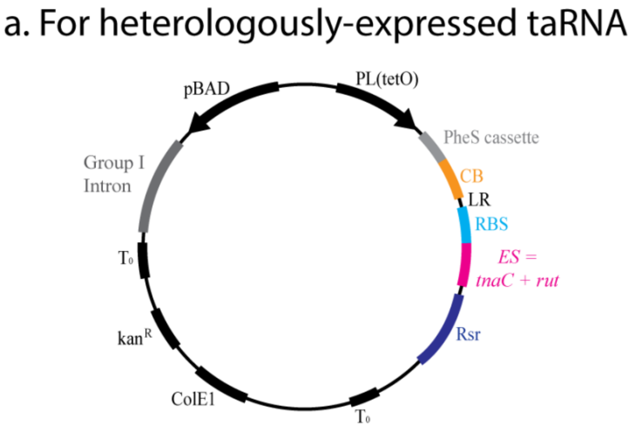

Supplementary Figure 1. INTERFACE plasmid maps (a). Plasmid map of O-INTERFACE (for heterologously expressed target RNAs). (b) Plasmid map INTERFACE (for native target RNAs).

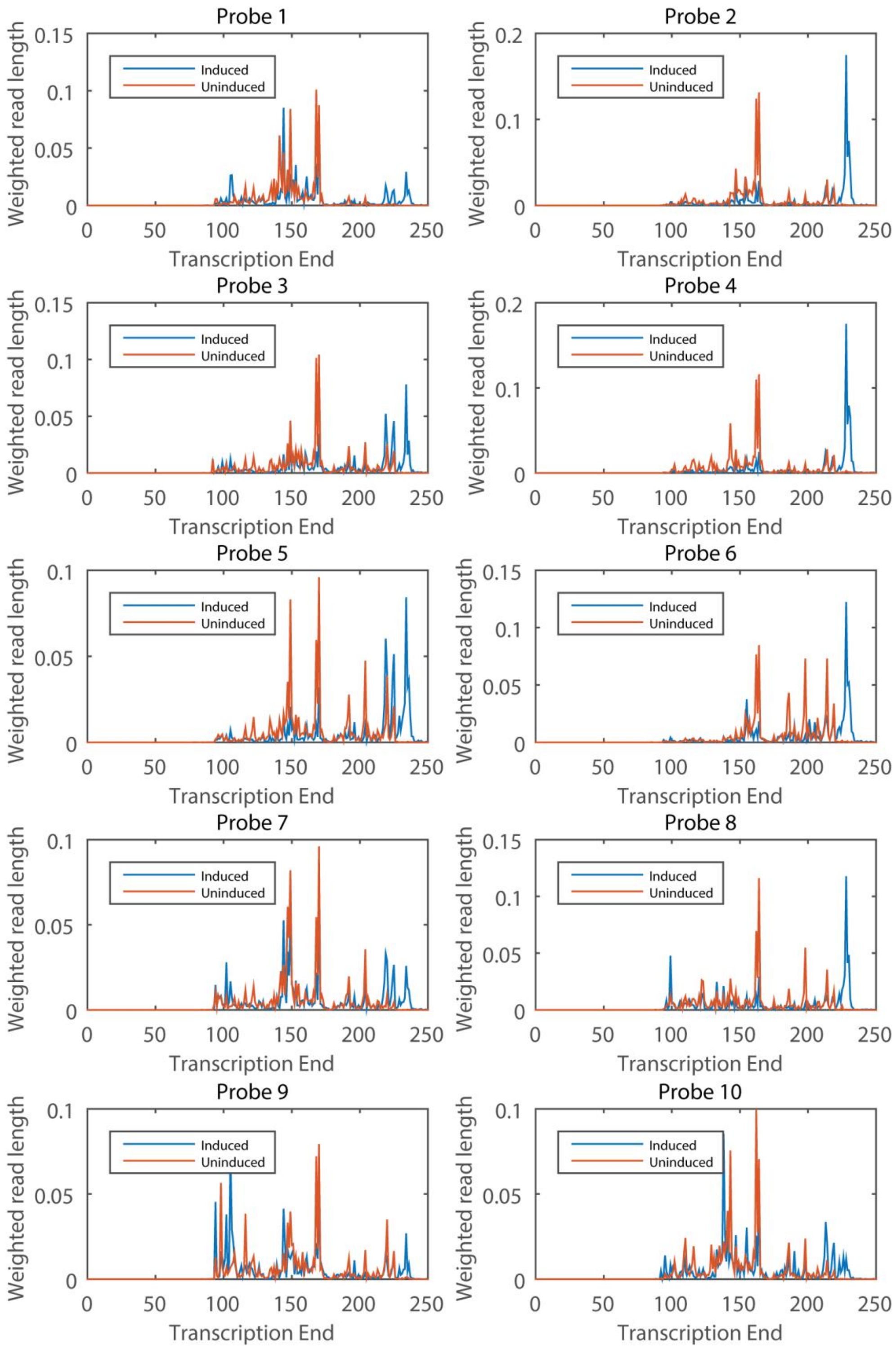
Probe 1
Probe 2
Probe 3
Probe 4
Probe 5
Probe 6
Probe 7
Probe 8
Probe 9
Probe 10
Induced
Uninduced
Weighted read length
Transcription End
0
50
100
150
200
250
0.05
0.1
0.15
0.2

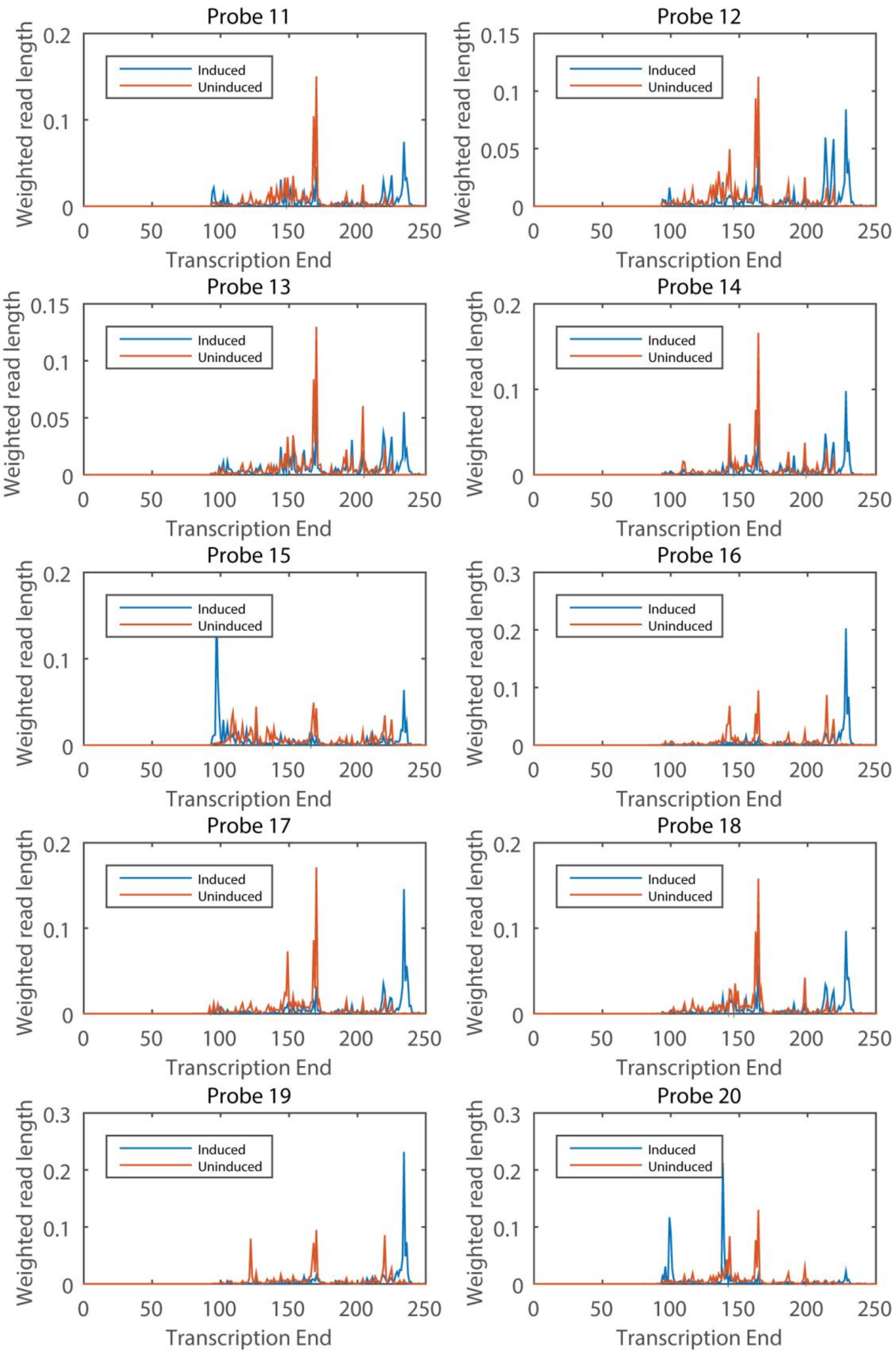

Probe 11
Probe 12
Probe 13
Probe 14
Probe 15
Probe 16
Probe 17
Probe 18
Probe 19
Probe 20
Induced
Uninduced
Weighted read length
Transcription End
0
50
100
150
200
250
0.05
0.1
0.15
0.2
0.3

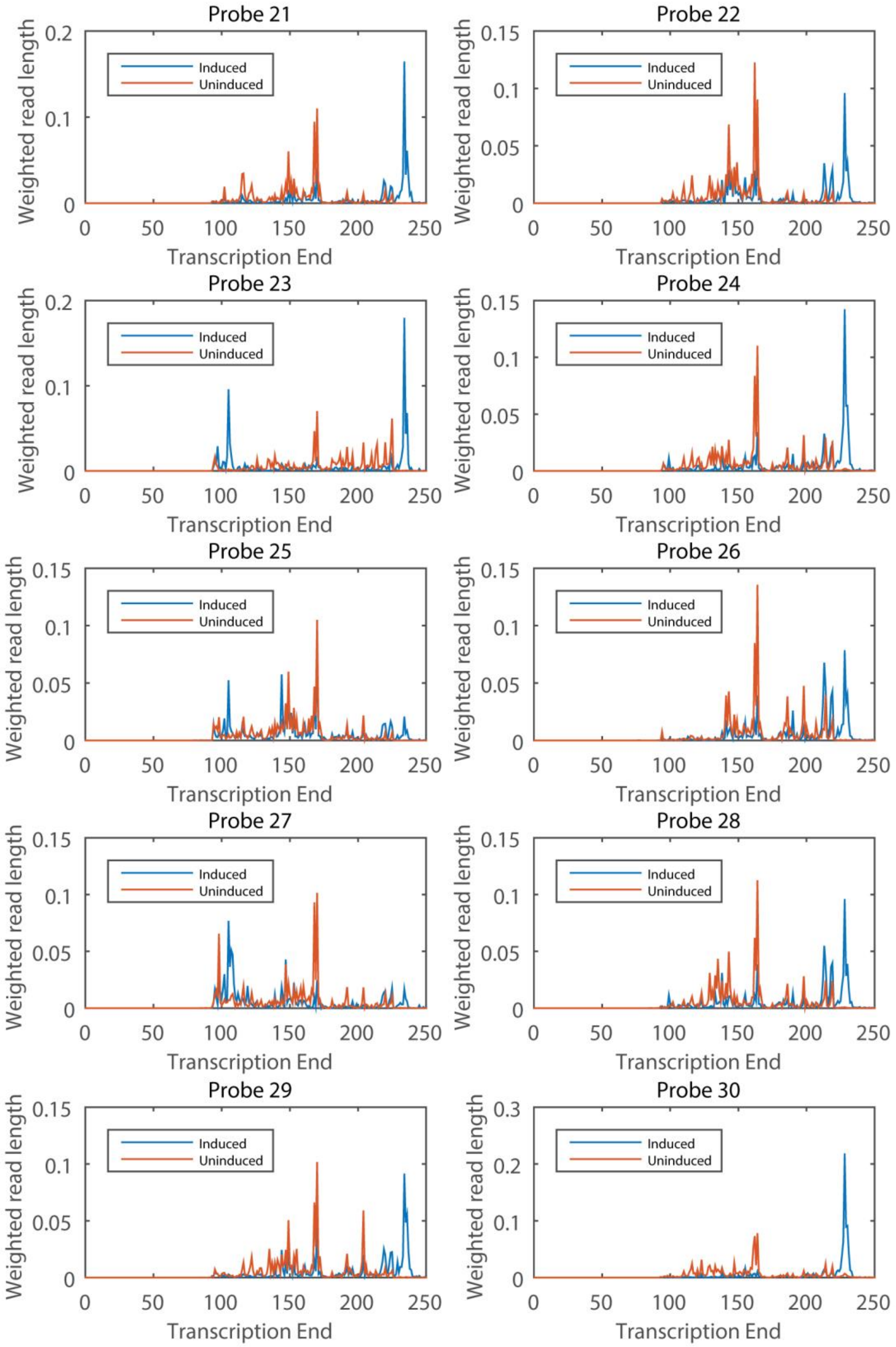

Supplementary Figure 2. O-INTERFACE transcription end traces for 30 new group I intron regions probed.

ArrS

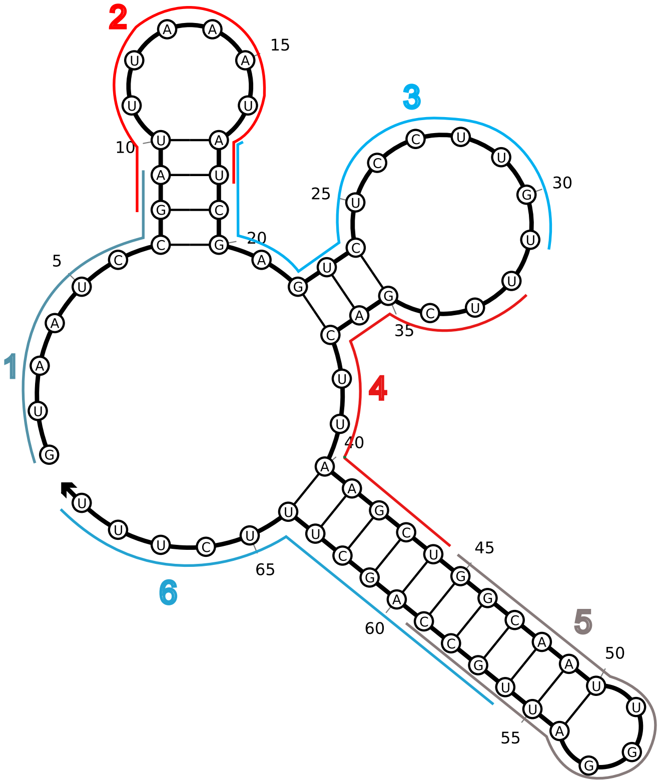

CyaR

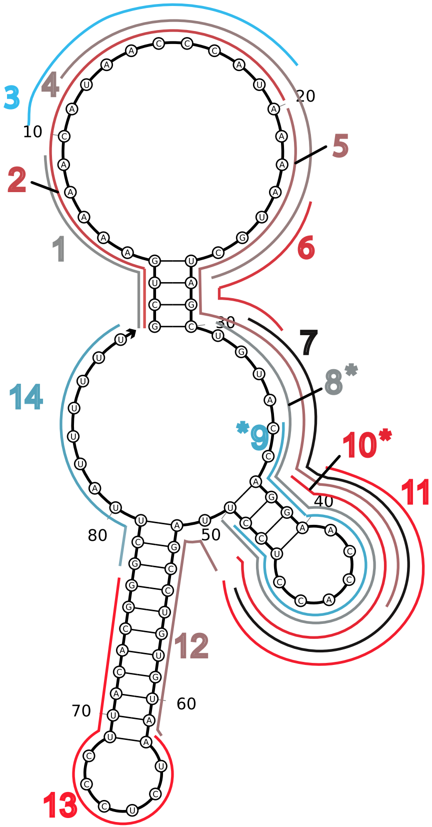

DicF

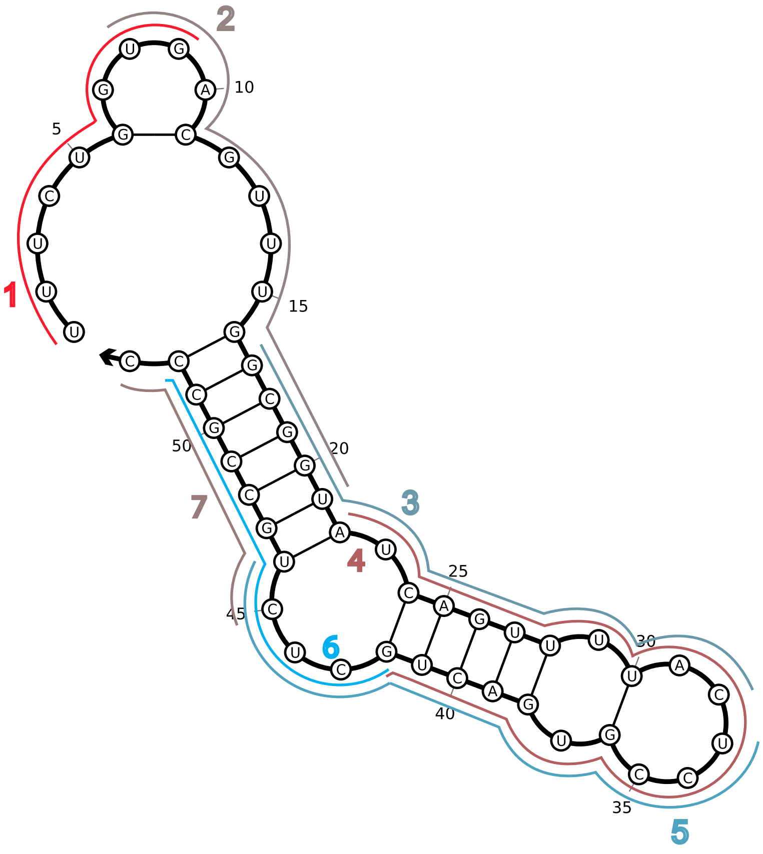

DsrA

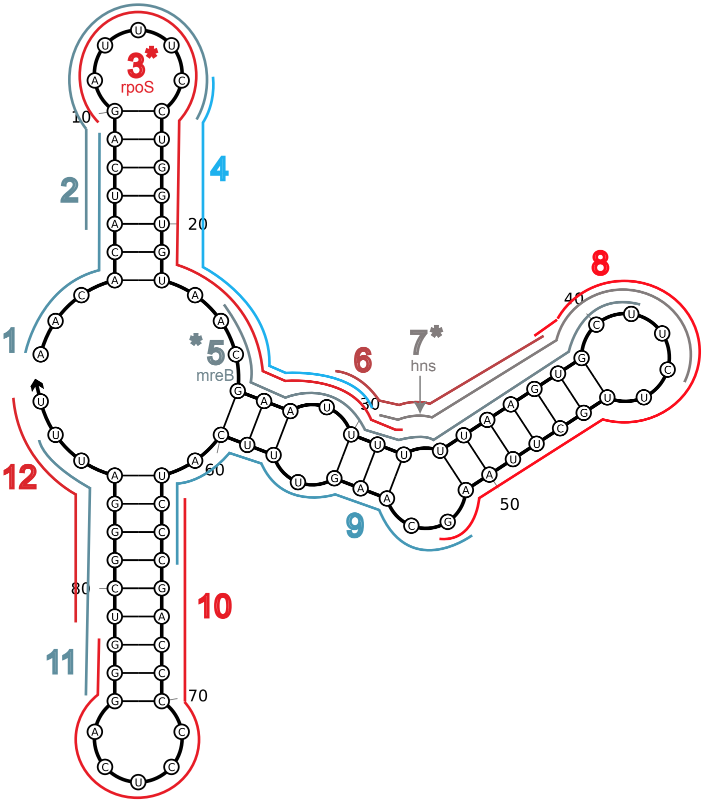

Ffs

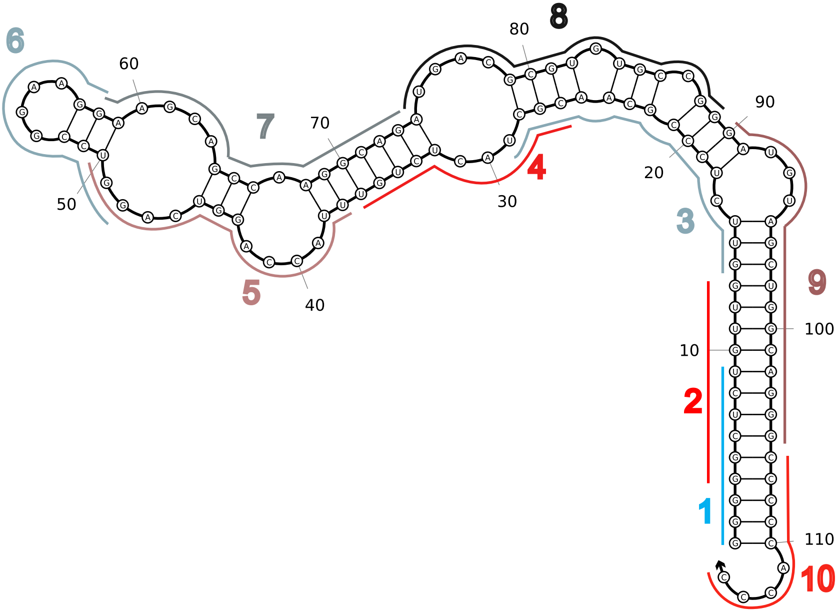

FnrS

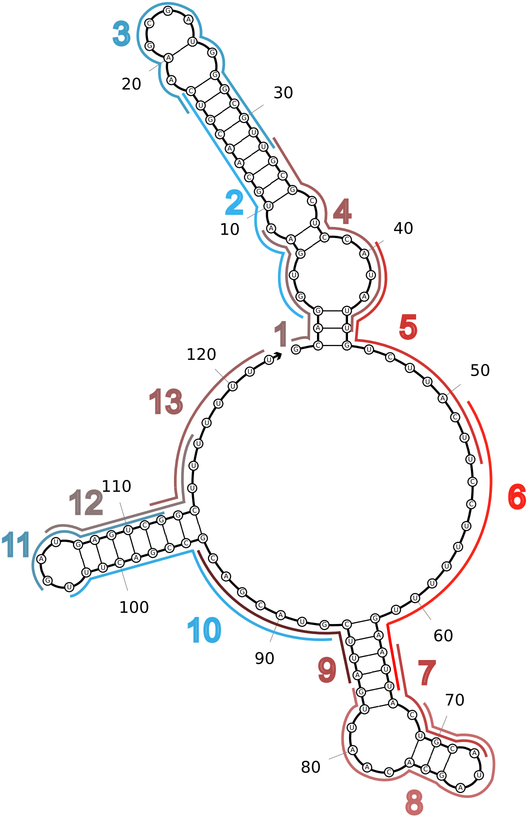

GadY

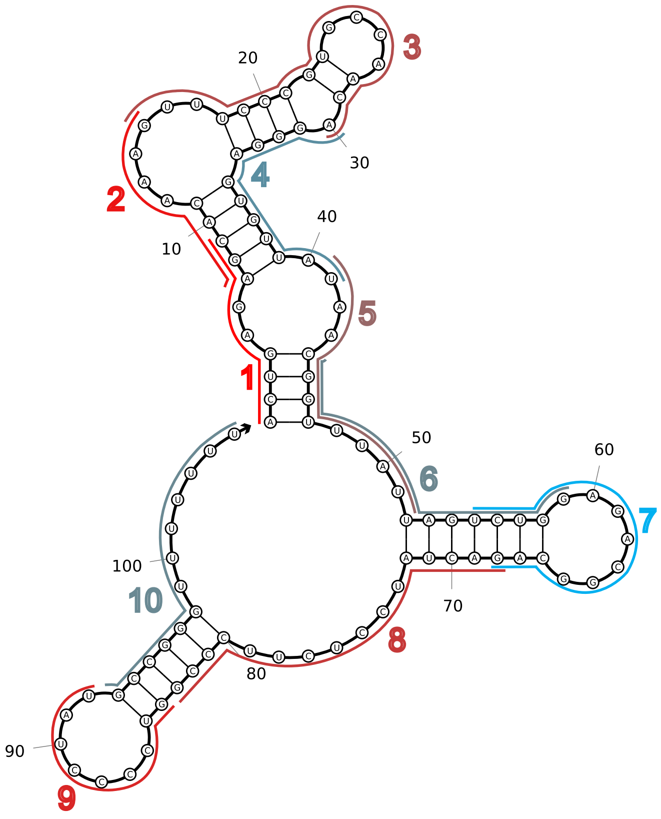

GcvB

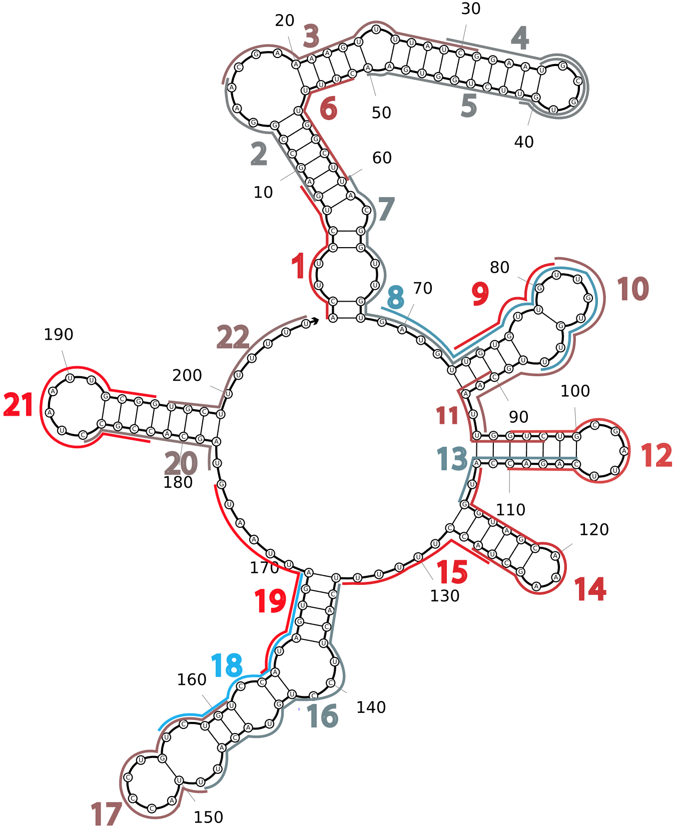

1
2
3
4
5
6
7
8
9
10
11
12
13
14
15
16
17
18
19
20
21
22
10
20
30
40
50
60
70
80
90
100
110
120
130
140
150
160
170
180
190
200

GlmY

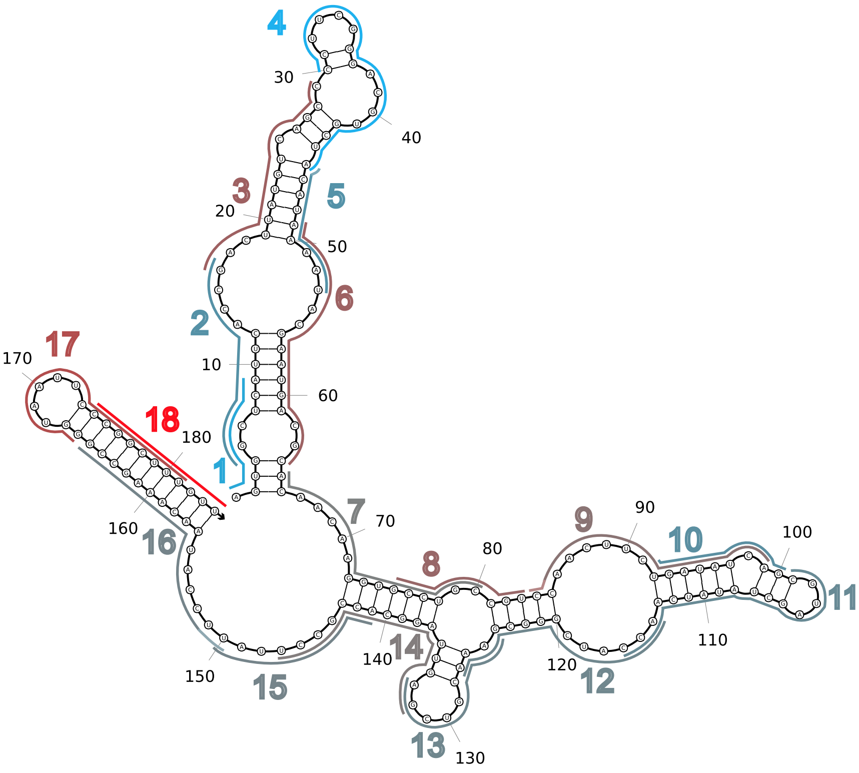

1
2
3
4
5
6
7
8
9
10
11
12
13
14
15
16
17
18
10
20
30
40
50
60
70
80
90
100
110
120
130
140
150
160
170
180

GlmZ

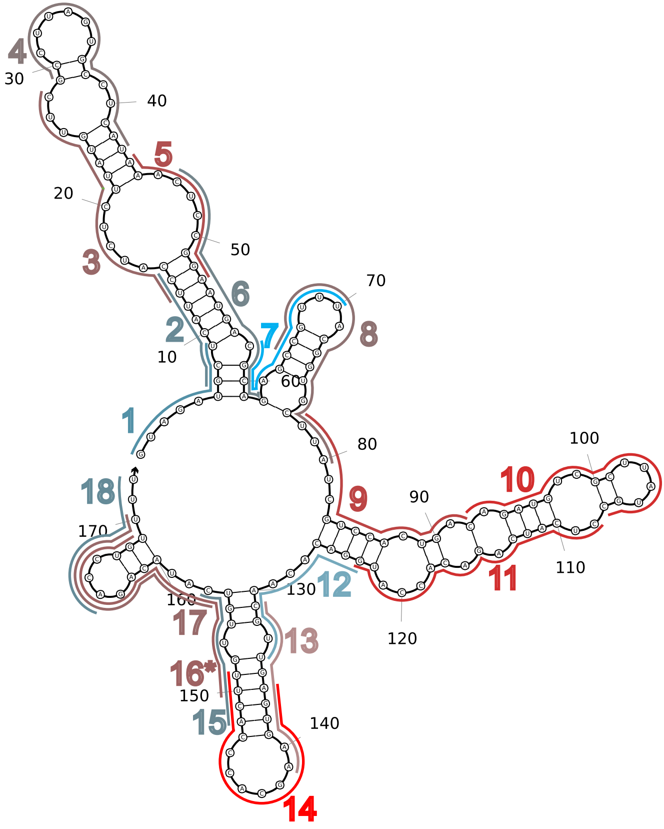
1
2
3
4
5
6
7
8
9
10
11
12
13
14
15
16*
17
18
10
20
30
40
50
60
70
80
90
100
110
120
130
140
150
160
170

InvR

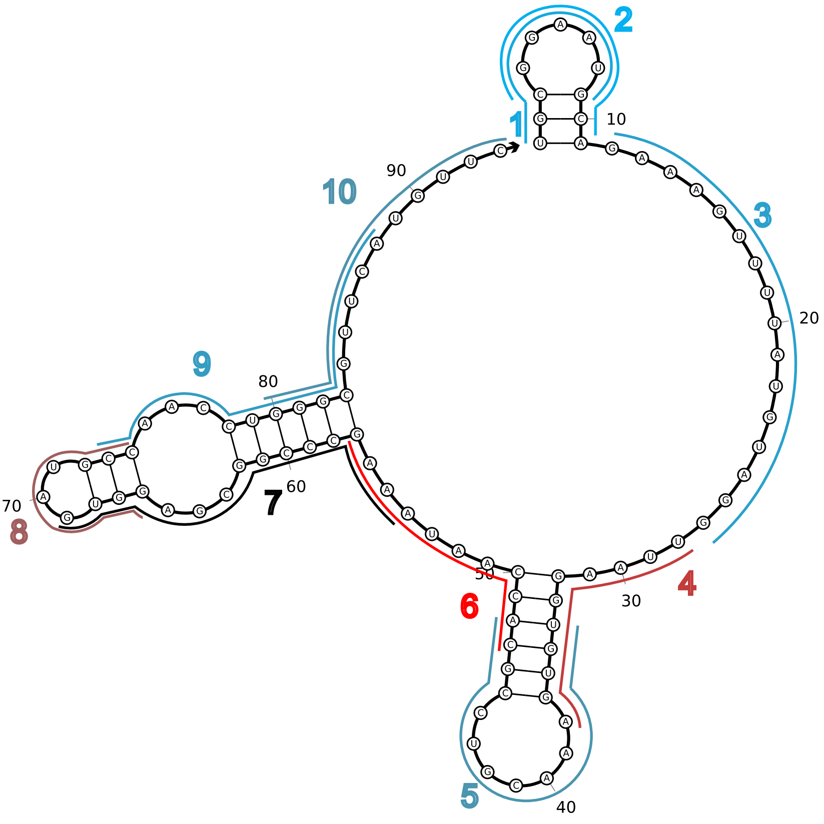

IpeX

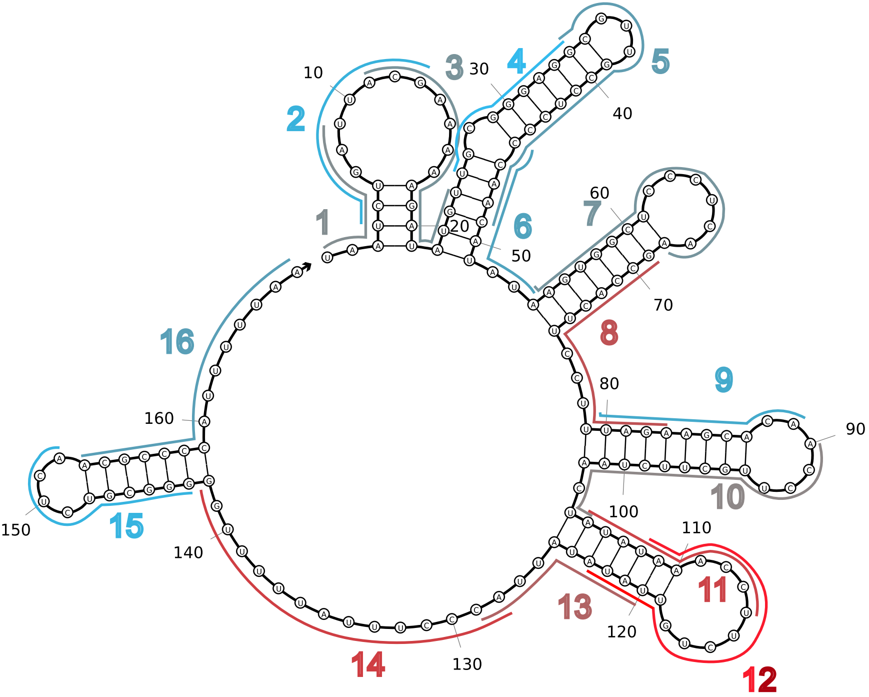
1
2
3
4
5
6
7
8
9
10
11
12
13
14
15
16
10
20
30
40
50
60
70
80
90
100
110
120
130
140
150
160

IsrB

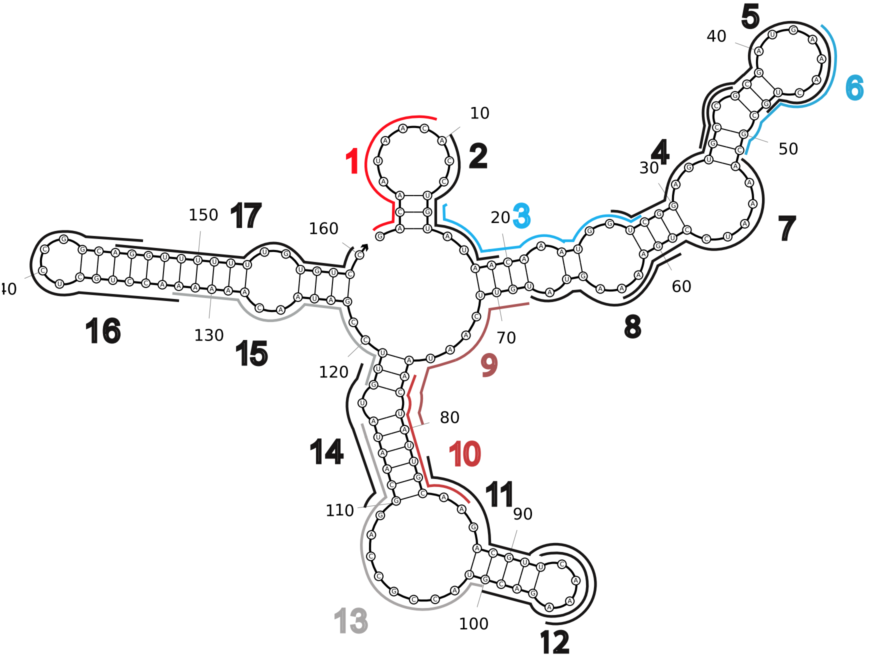
1
2
3
4
5
6
7
8
9
10
11
12
13
14
15
16
17
10
20
30
40
50
60
70
80
90
100
110
120
130
150
160

IsrC

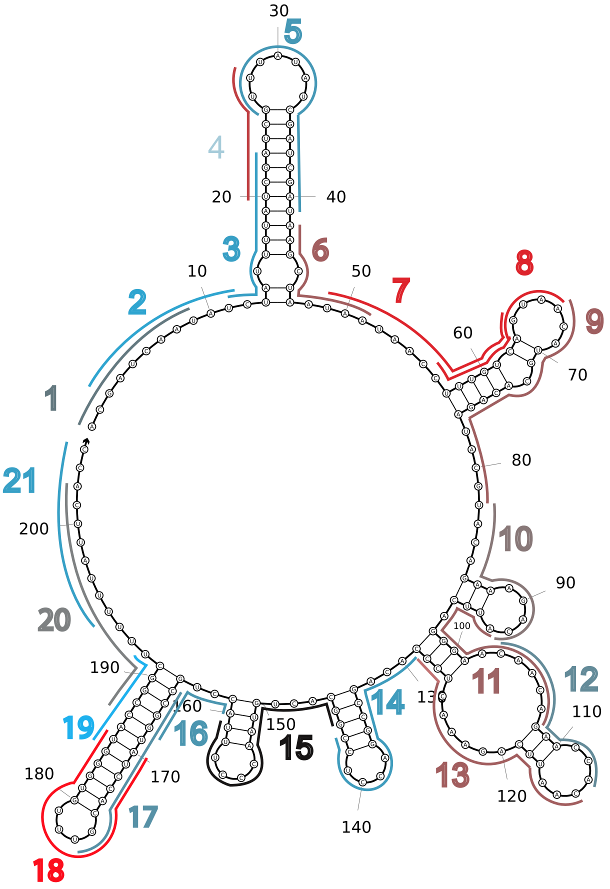

1
2
3
4
5
6
7
8
9
10
11
12
13
14
15
16
17
18
19
20
21
10
20
30
40
50
60
70
80
90
100
110
120
130
140
150
160
170
180
190
200

IstR-1

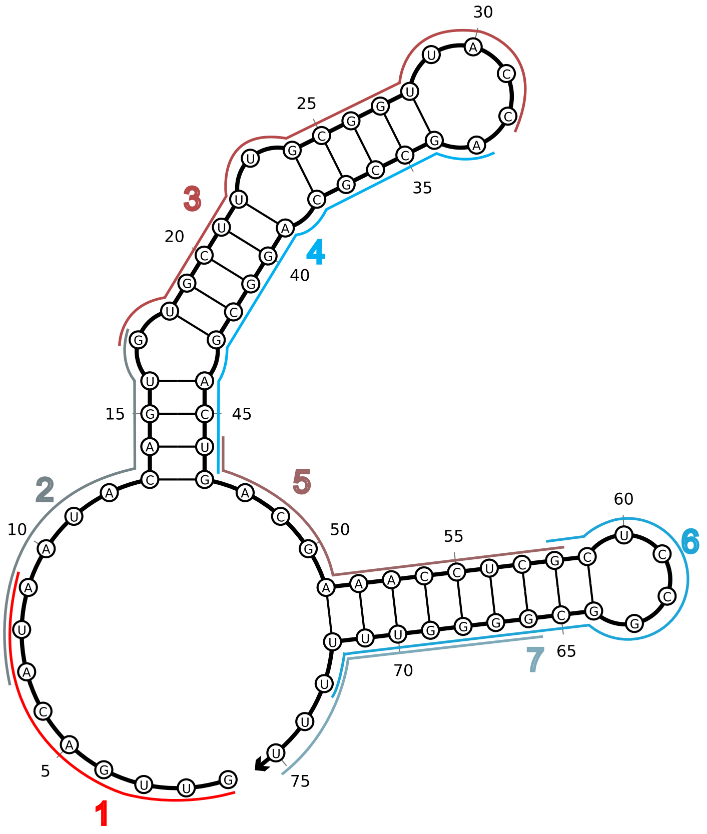

McaS

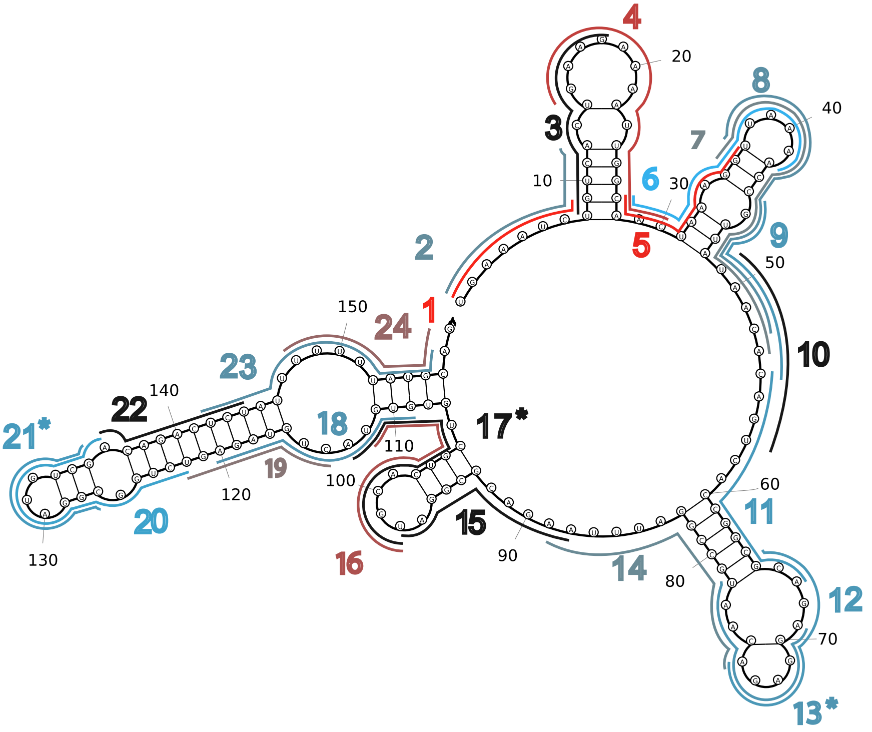
1
2
3
4
5
6
7
8
9
10
11
12
13*
14
15
16
17*
18
19
20
21*
22
23
24
10
20
30
40
50
60
70
80
90
100
110
120
130
140
150

MgrR

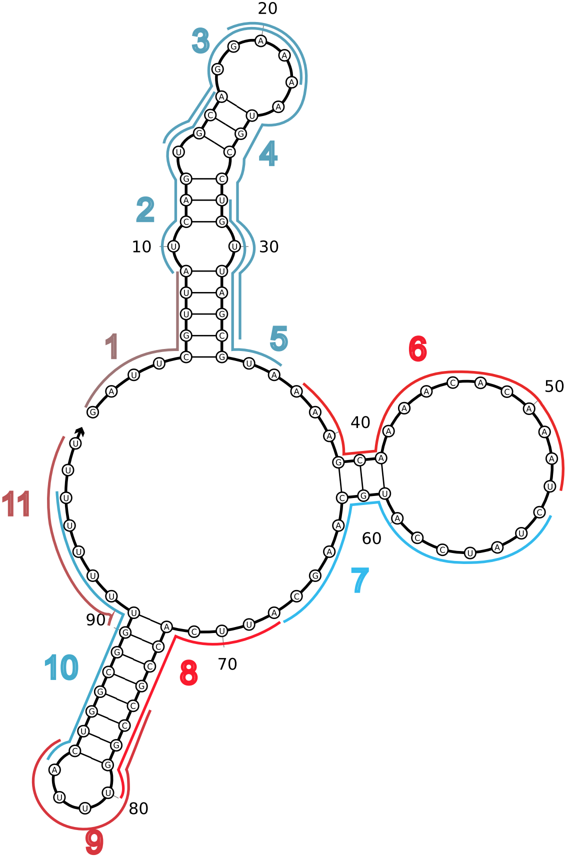

MicC

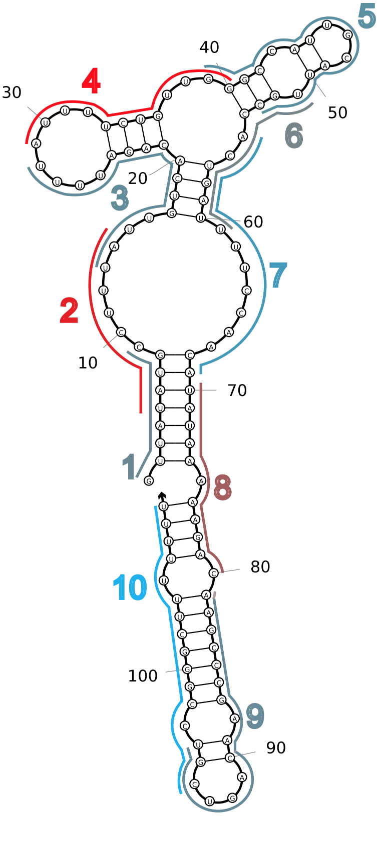

MicF

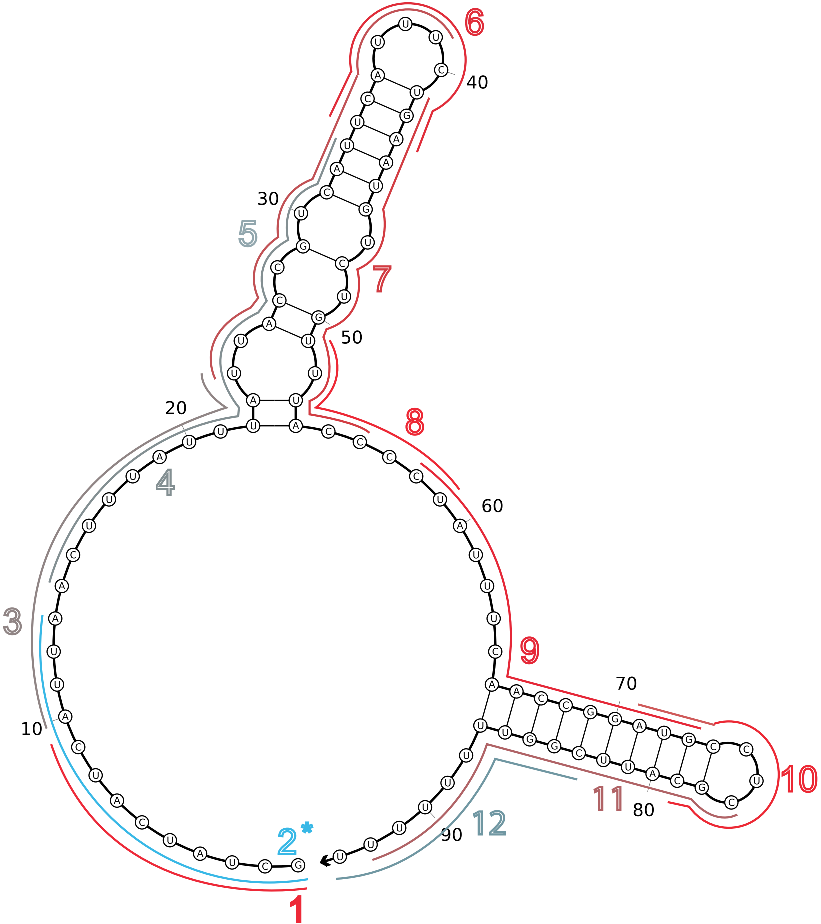

MicL

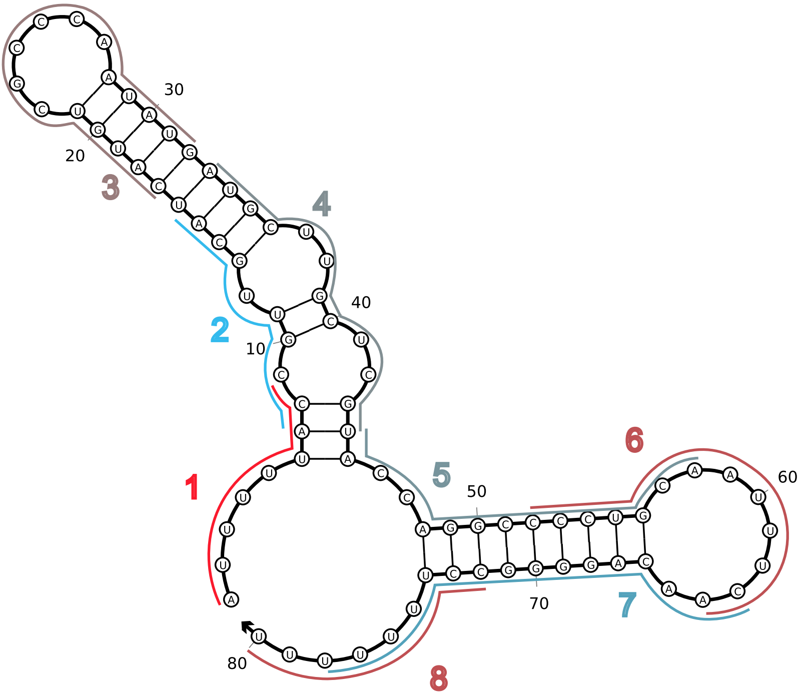

Nc2

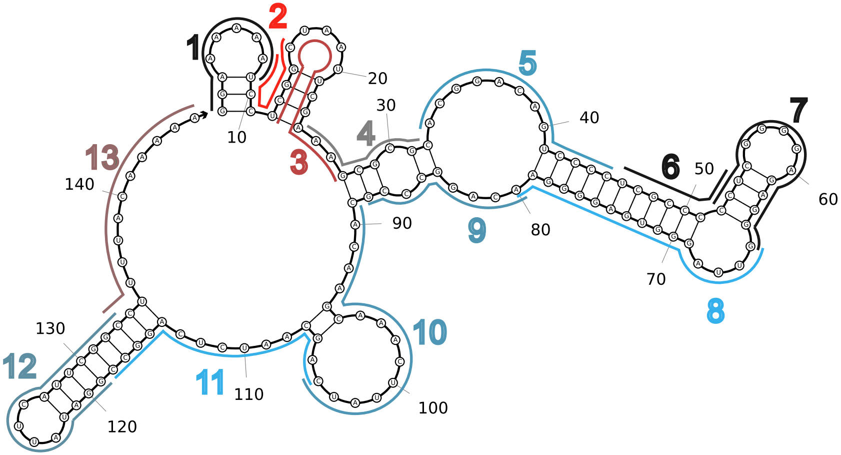
1
2
3
4
5
6
7
8
9
10
11
12
13
10
20
30
40
50
60
70
80
90
100
110
120
130
140

Nc5

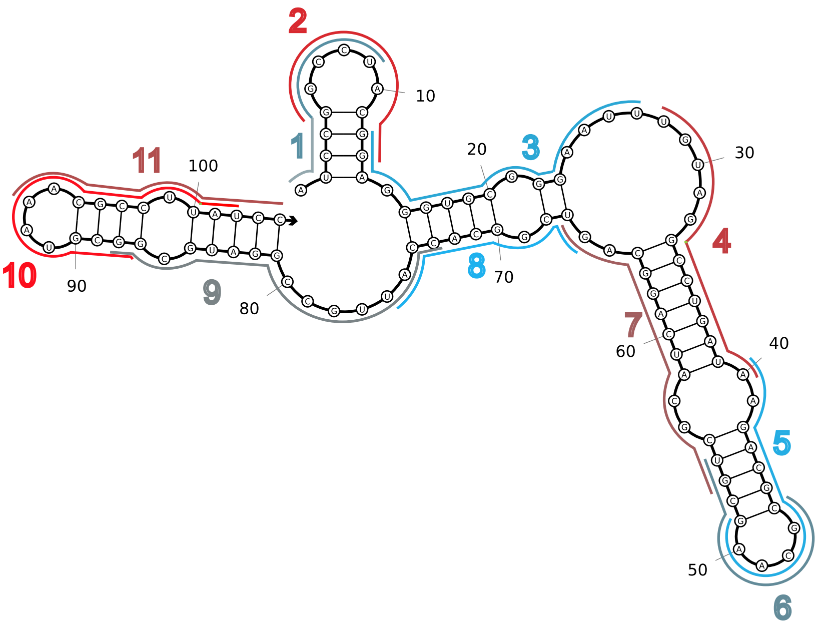

OmrA

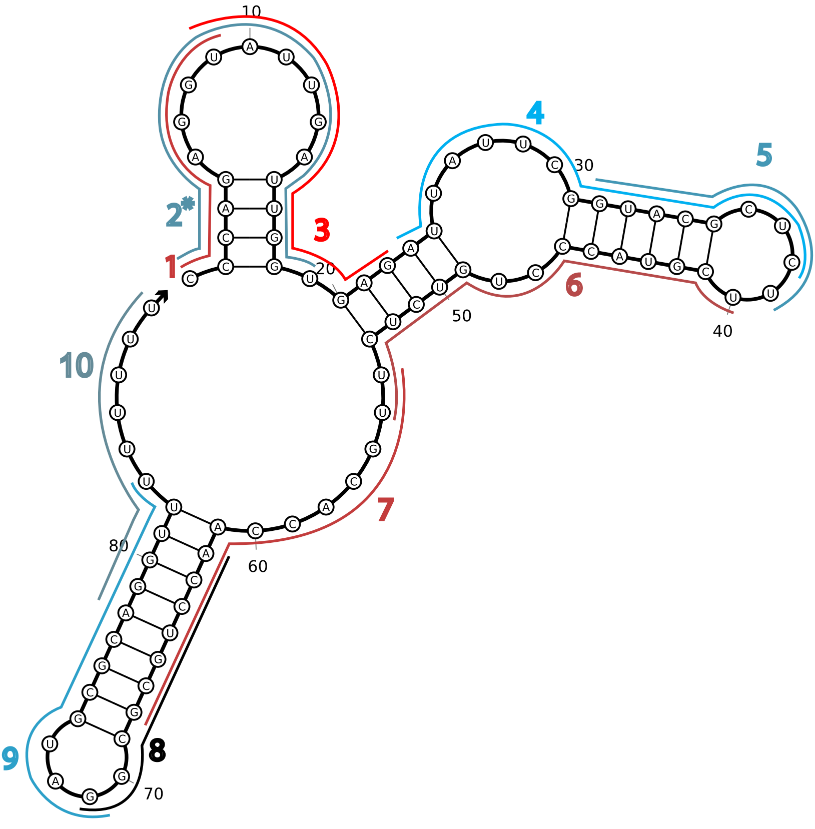

OmrB

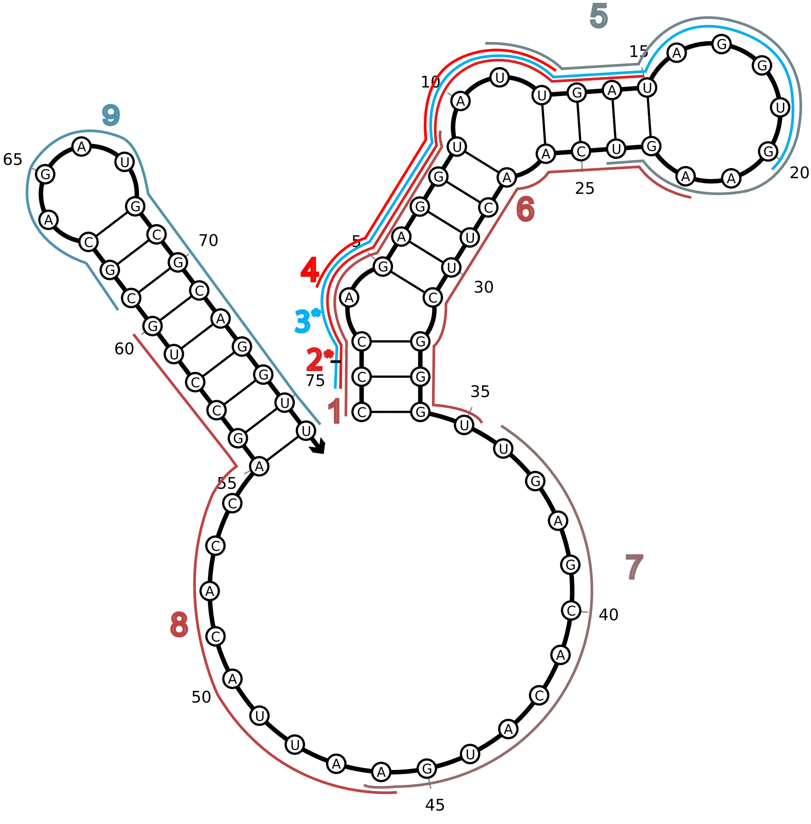

OxyS

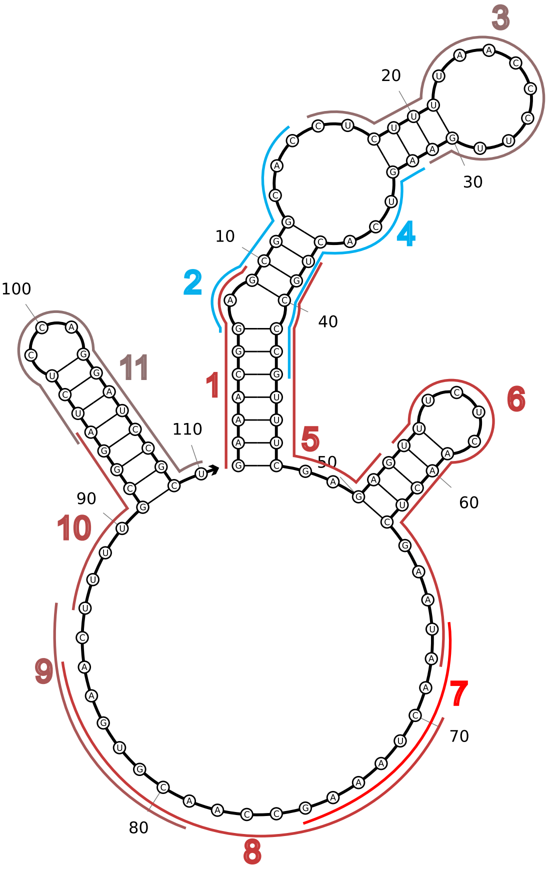

PsrD

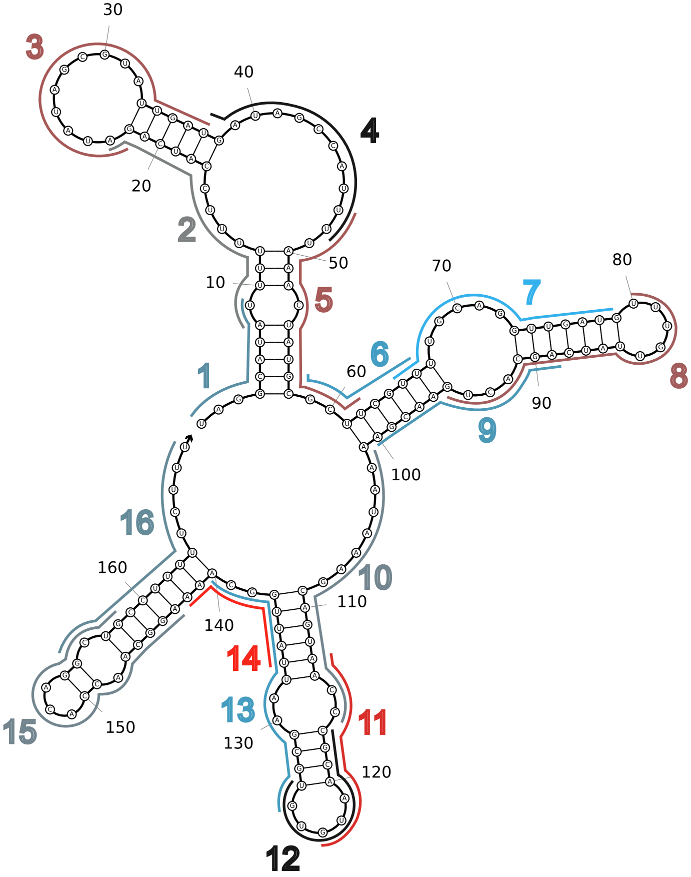

30
3
40
4
20
2
10
50
5
70
7
80
6
60
1
8
90
9
100
16
160
10
110
140
14
13
11
15
150
130
120
12

PsrN

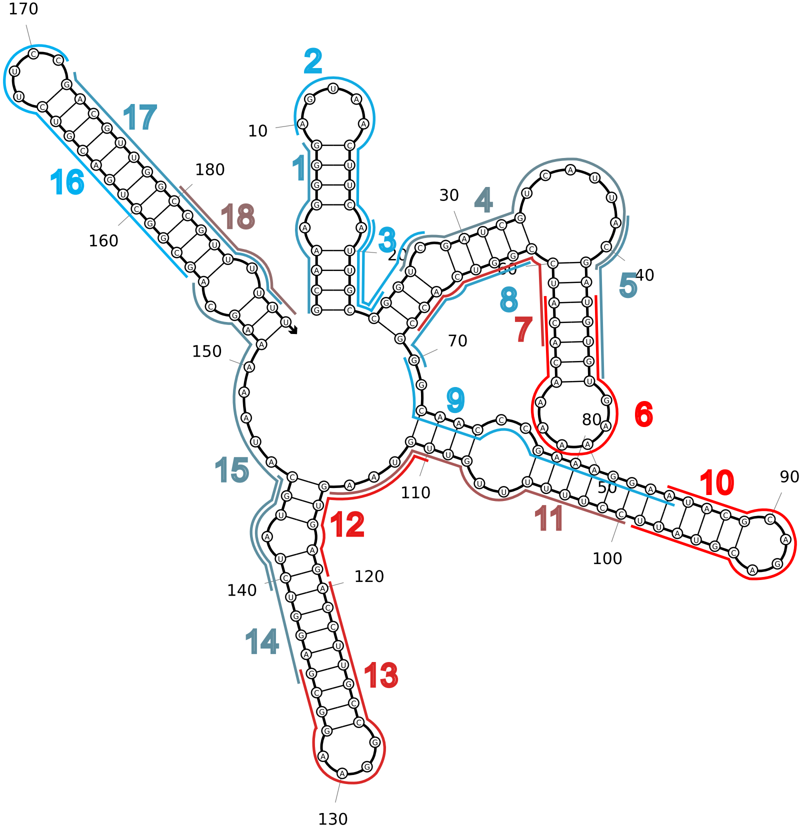

1
2
3
4
5
6
7
8
9
10
11
12
13
14
15
16
17
18
10
20
30
40
50
60
70
80
90
100
110
120
130
140
150
160
170
180

RdlA

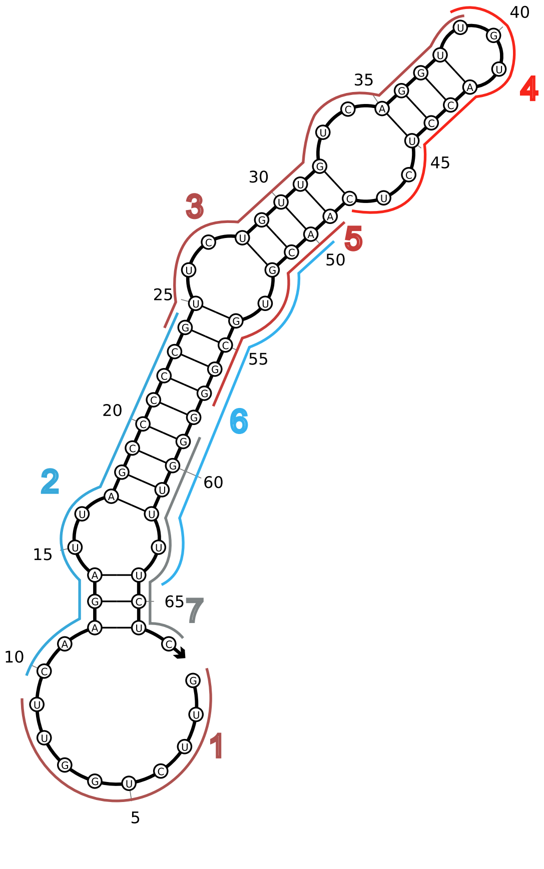

RdlB

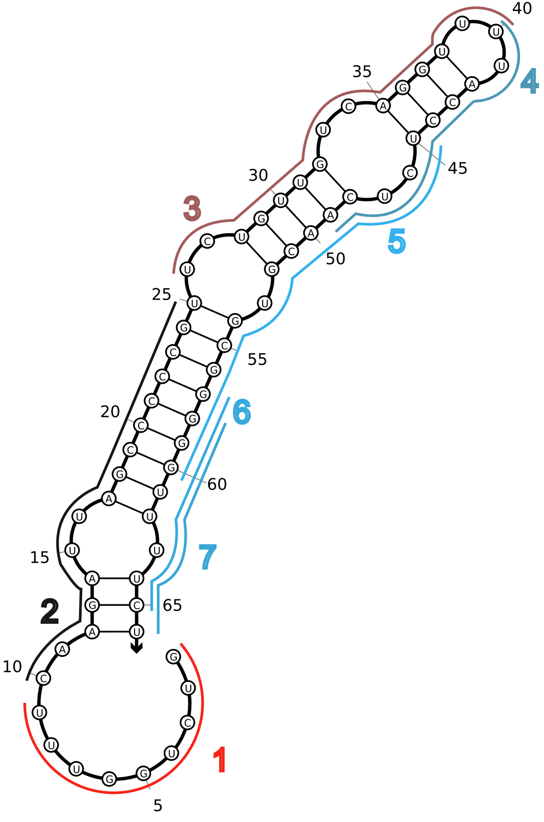

RdlC

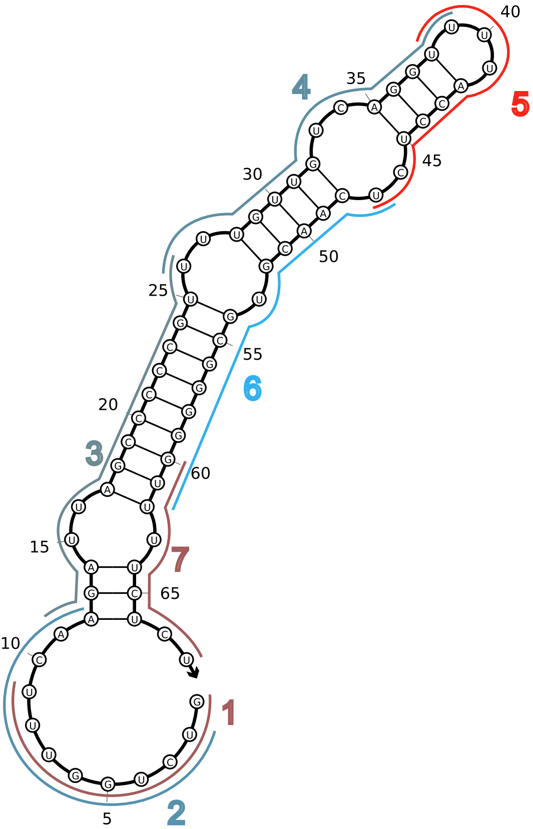

RdlD

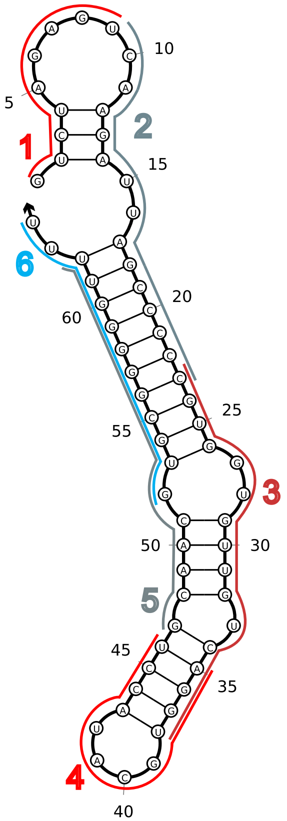

RprA

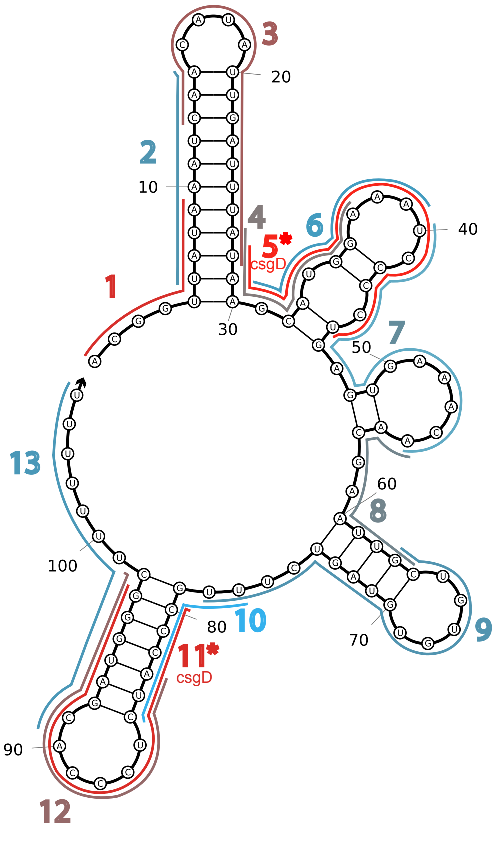

RseX

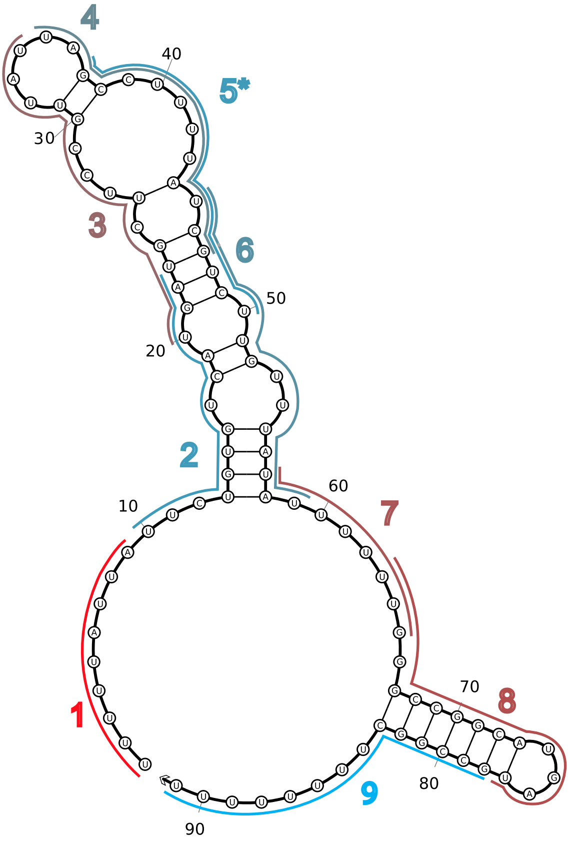

RybA (MntS)

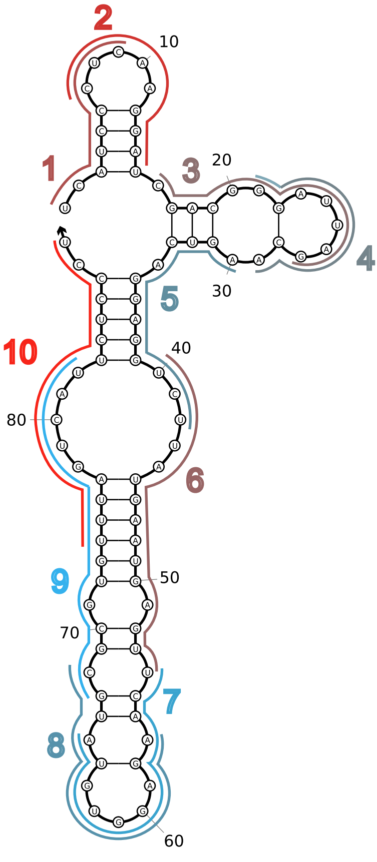

RybB

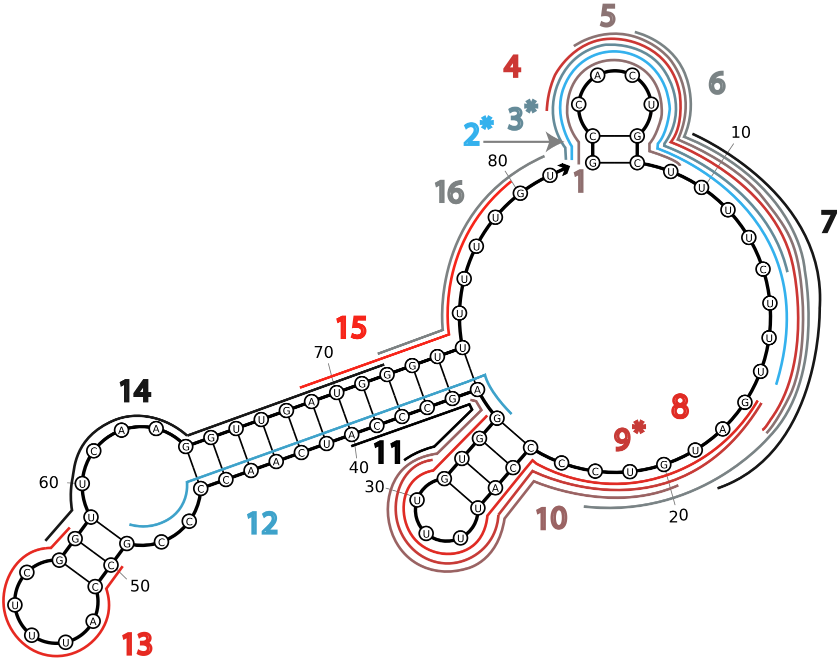

RydB

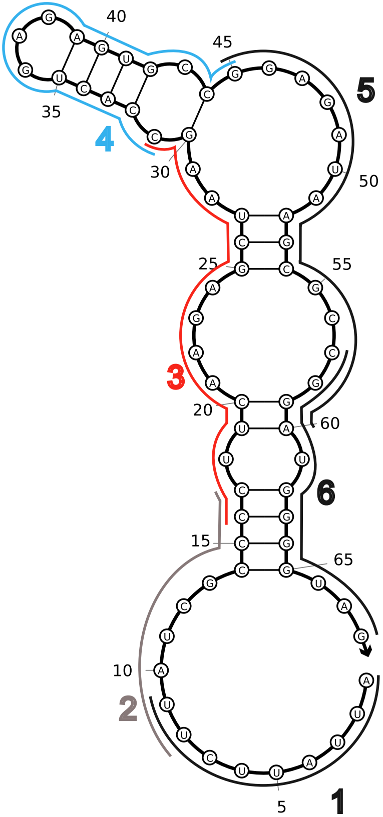

RydC

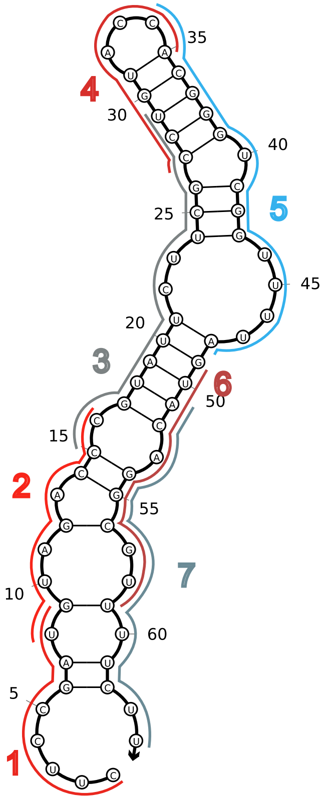

RyeA

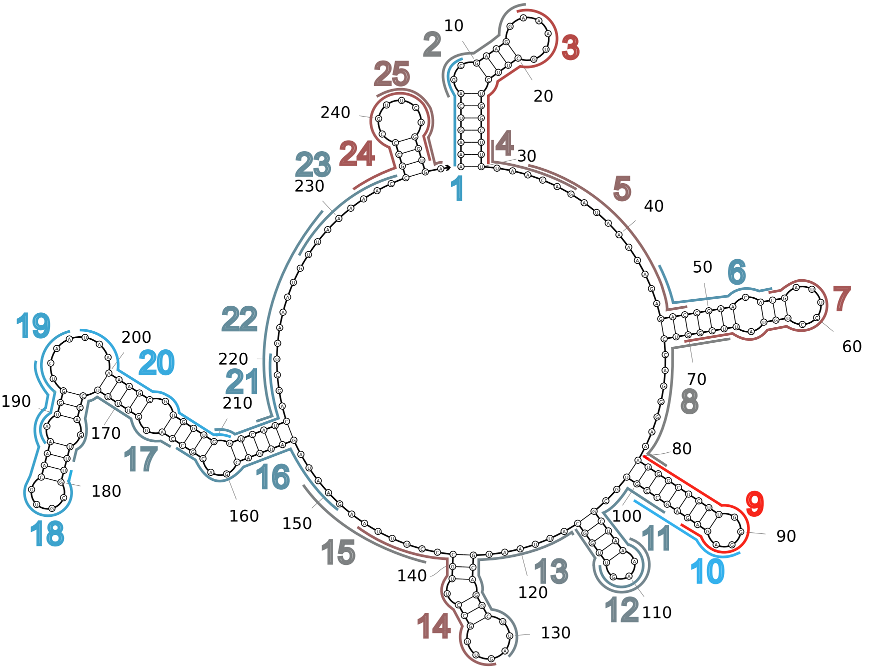
1
2
3
4
5
6
7
8
9
10
11
12
13
14
15
16
17
18
19
20
21
22
23
24
25
10
20
30
40
50
60
70
80
90
100
110
120
130
140
150
160
170
180
190
200
210
220
230
240

RyeB

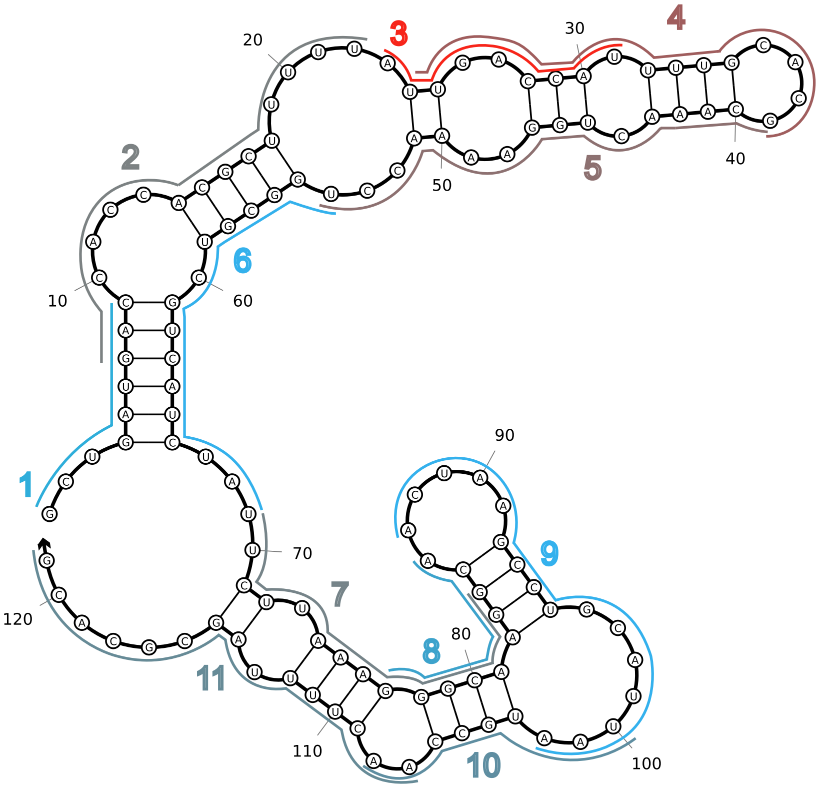

RyeF

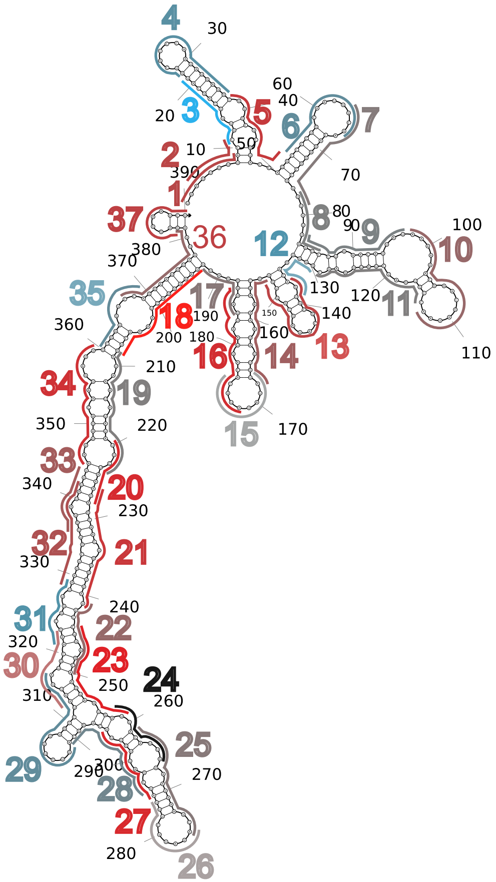

1
2
3
4
5
6
7
8
9
10
11
12
13
14
15
16
17
18
19
20
21
22
23
24
25
26
27
28
29
30
31
32
33
34
35
36
37
10
20
30
40
50
60
70
80
90
100
110
120
130
140
150
160
170
180
190
200
210
220
230
240
250
260
270
280
290
300
310
320
330
340
350
360
370
380
390

RyfA

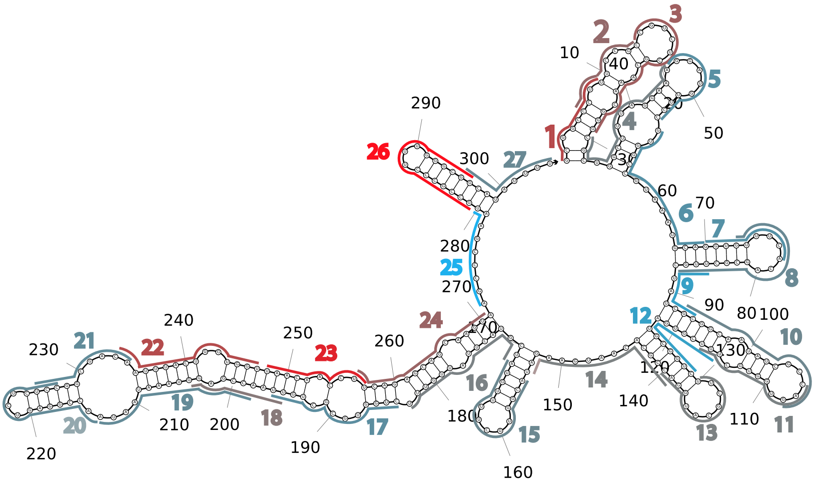

1
2
3
4
5
6
7
8
9
10
11
12
13
14
15
16
17
18
19
20
21
22
23
24
25
26
27
10
40
50
60
70
80
90
100
110
120
130
140
150
160
180
190
200
210
220
230
240
250
260
270
280
290
300

RyfB

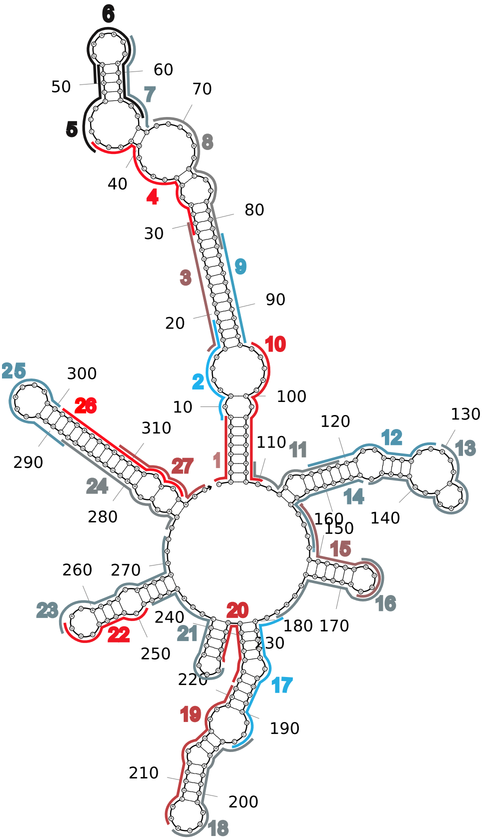

6
60
50
7
70
5
8
40
4
80
30
9
3
90
20
10
2
100
10
25
300
26
310
120
130
110
11
12
13
1
27
290
24
14
280
160
150
140
15
260
270
16
23
240
20
180
170
22
21
230
250
220
17
19
190
210
200
18

RyfC

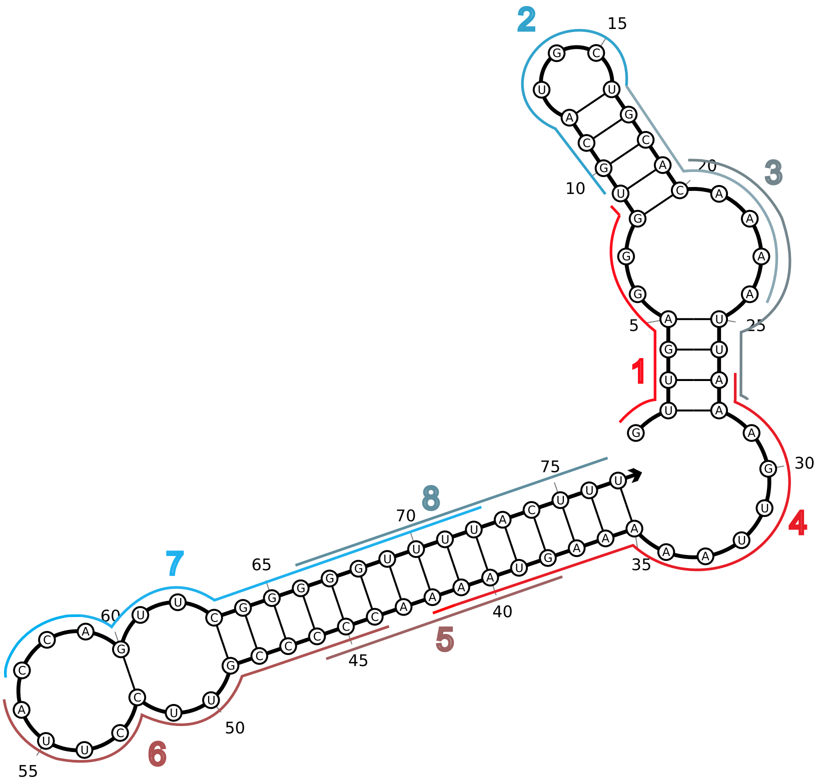

RyfD

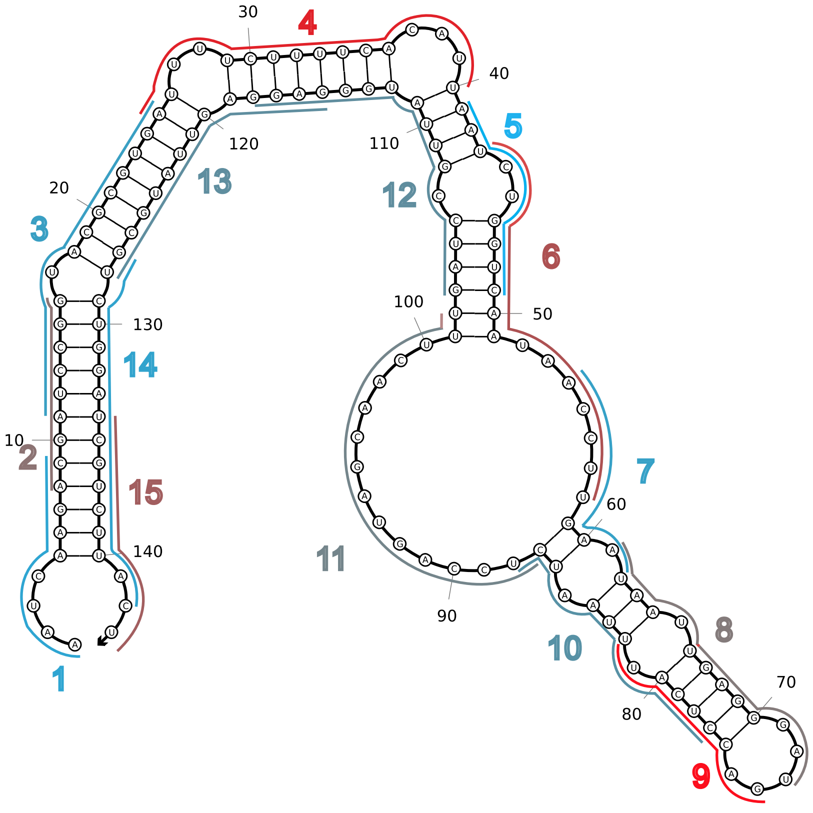

1
2
3
4
5
6
7
8
9
10
11
12
13
14
15
10
20
30
40
50
60
70
80
90
100
110
120
130
140

RygC

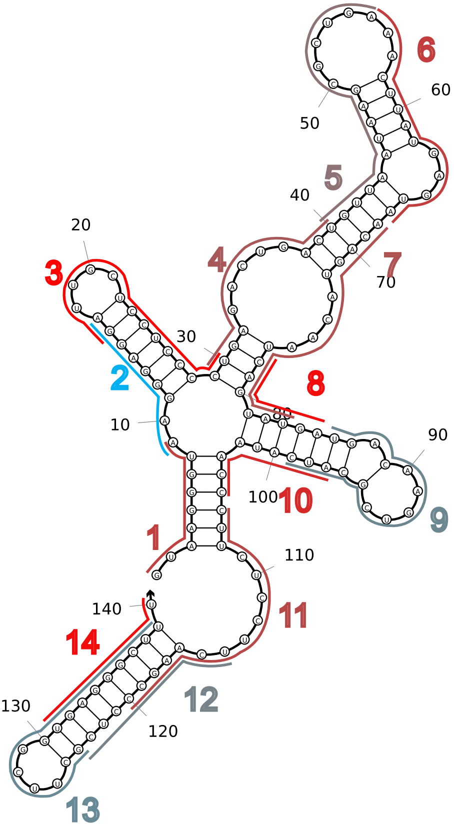

1
2
3
4
5
6
7
8
9
10
11
12
13
14
10
20
30
40
50
60
70
90
100
110
120
130
140

RygD (SibD)

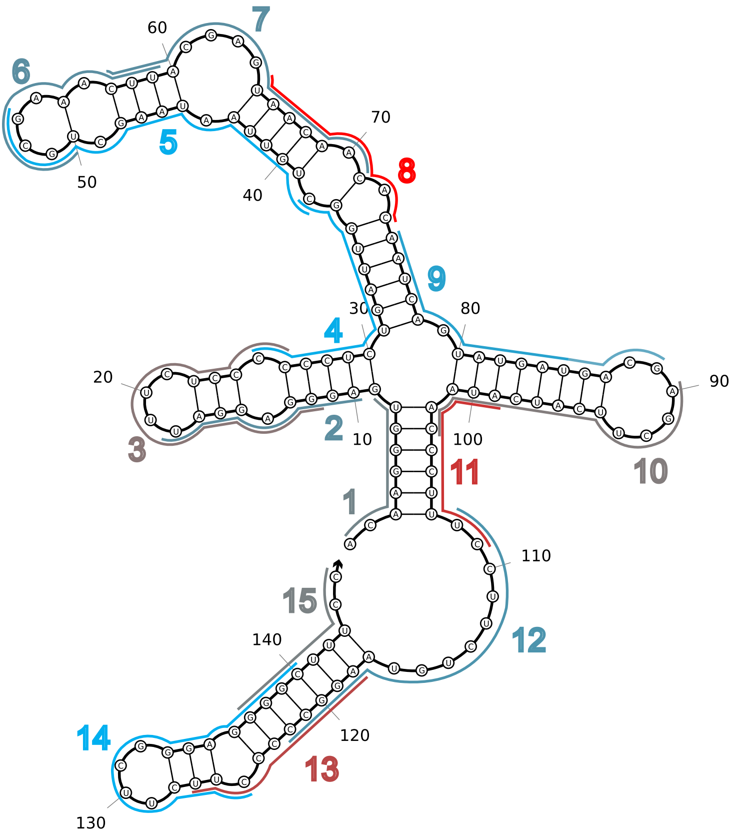
1
2
3
4
5
6
7
8
9
10
11
12
13
14
15
10
20
30
40
50
60
70
80
90
100
110
120
130
140

RyhB

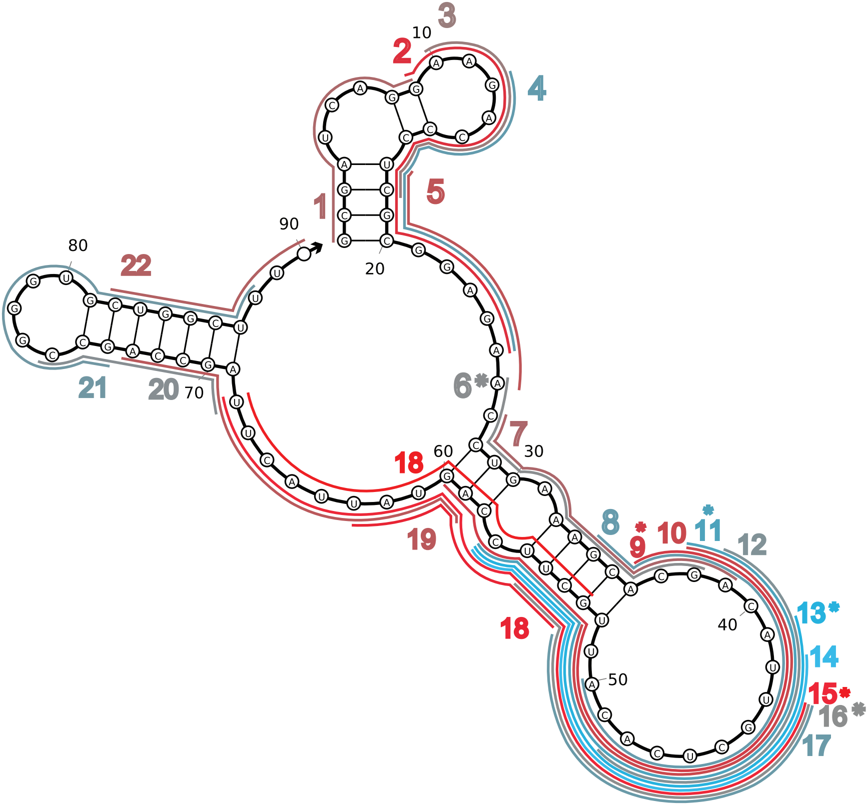

1
2
3
4
5
6*
7
8
9*
10
11*
12
13*
14
15*
16*
17
18
19
20
21
22
10
20
30
40
50
60
70
80
90

RyjB

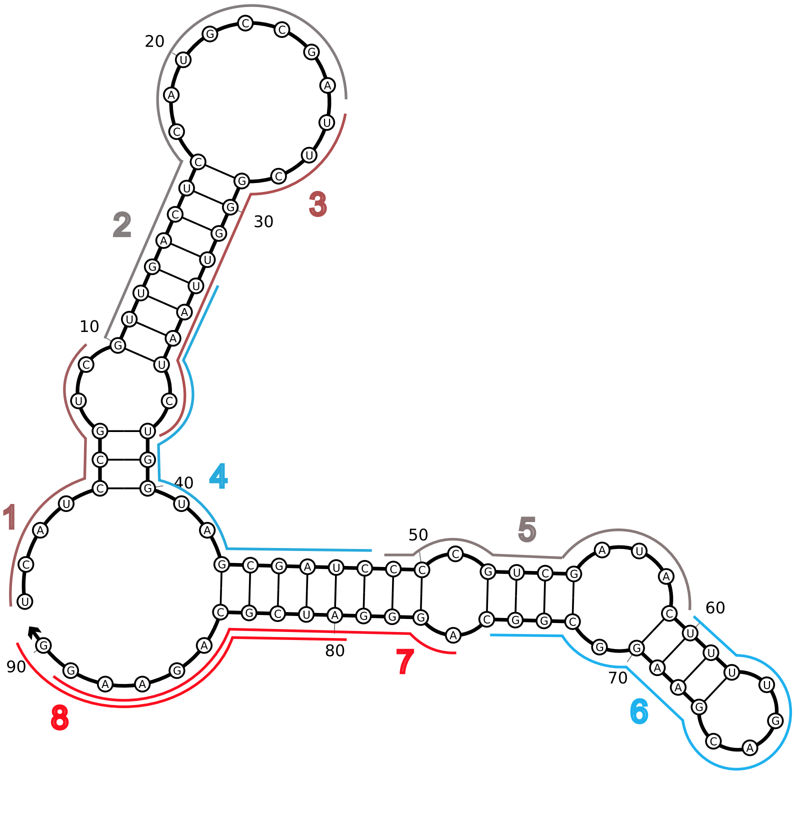

SrgS

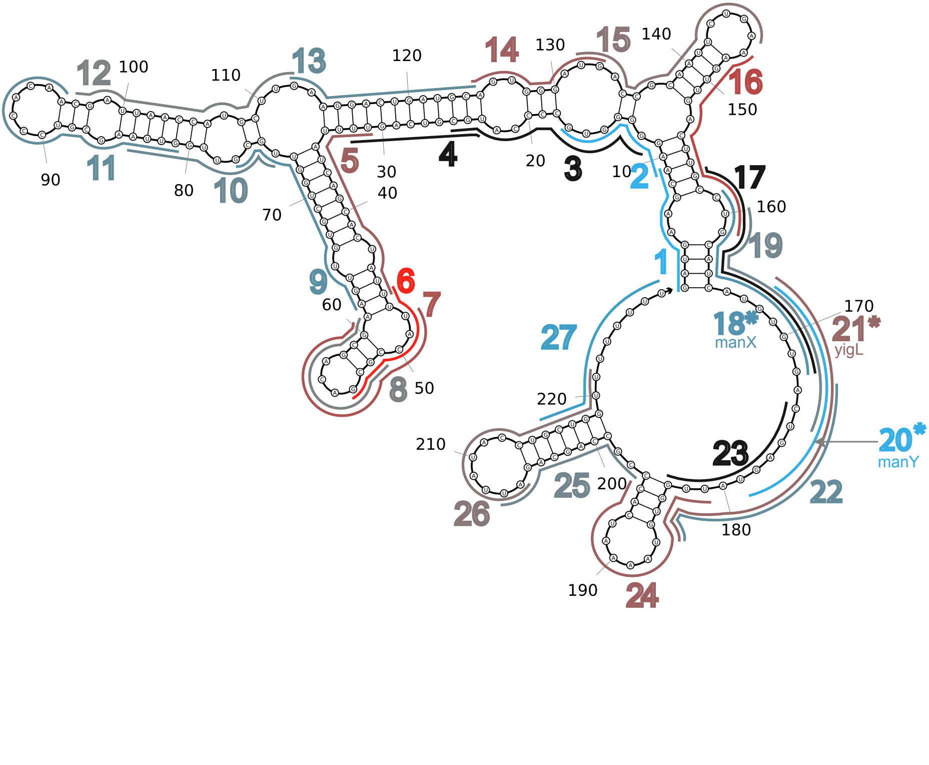
1
2
3
4
5
6
7
8
9
10
11
12
13
14
15
16
17
18*
manX
19
20*
manY
21*
yigL
22
23
24
25
26
27
10
20
30
40
50
60
70
80
90
100
110
120
130
140
150
160
170
180
190
200
210
220

SibA

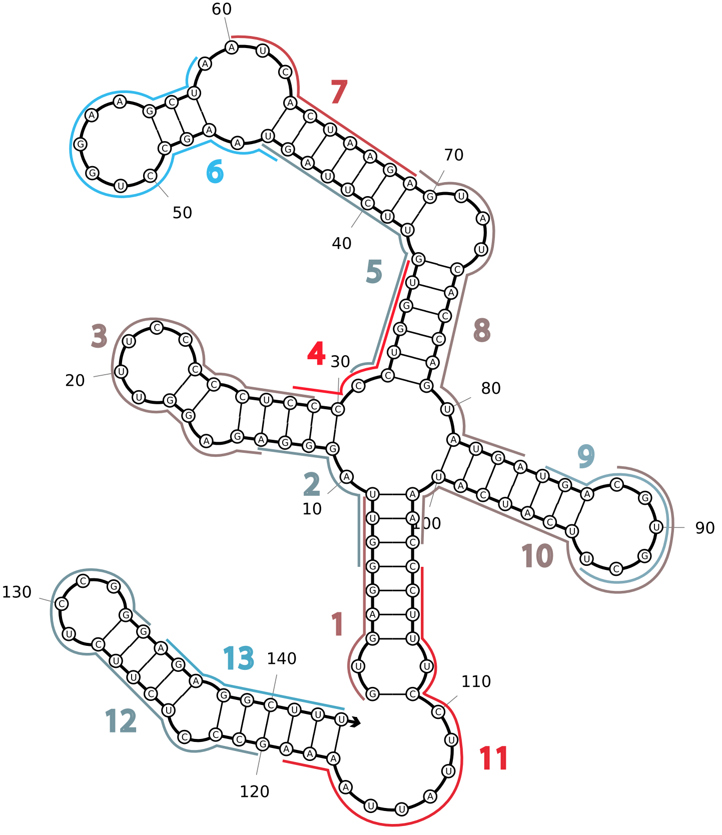

60
7
70
6
50
40
5
8
3
20
4
30
80
9
90
2
10
100
10
130
1
13
140
110
12
11
120

sibB

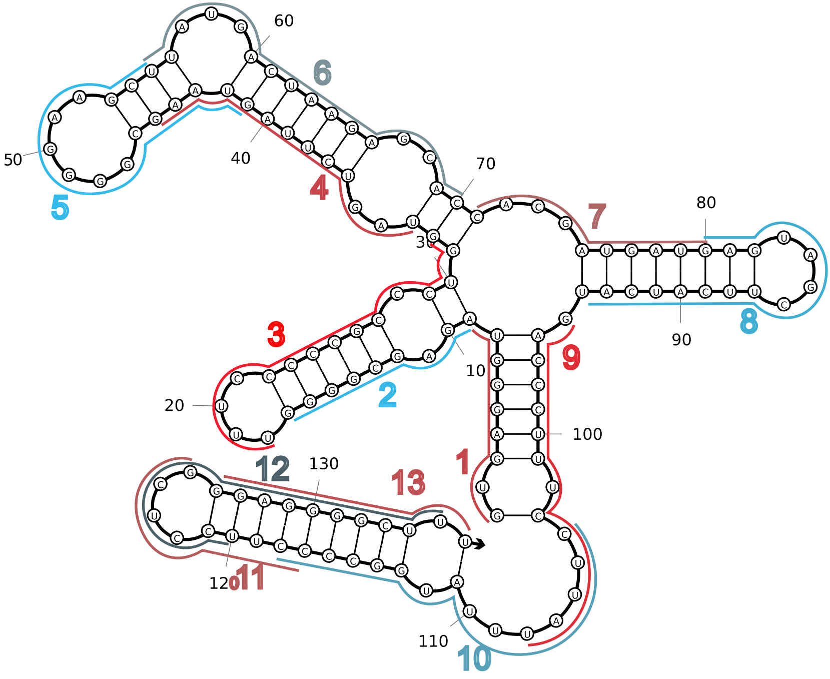

SibE

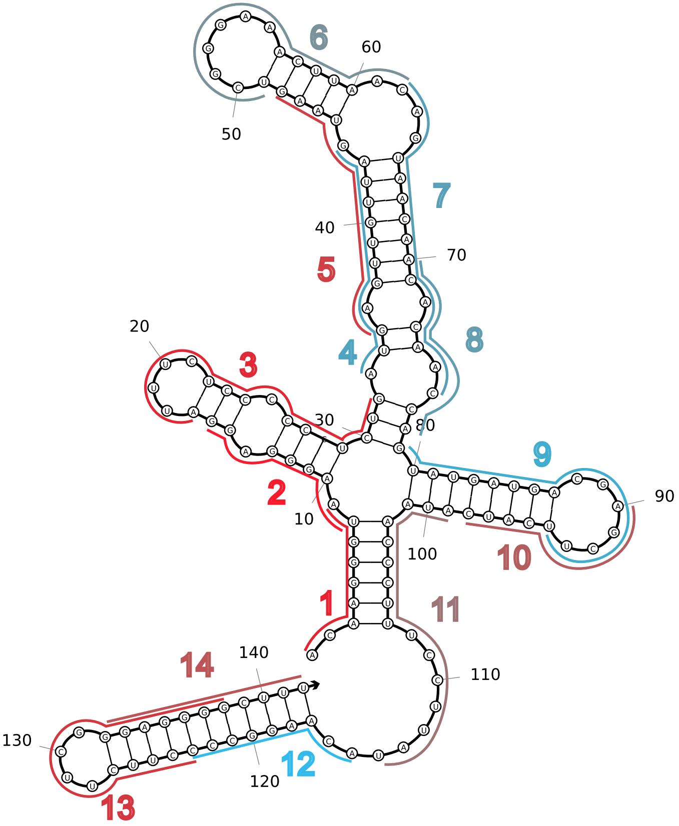

6
60
50
7
40
70
5
20
3
8
4
30
80
9
2
90
10
100
10
1
11
14
140
110
130
12
120
13

SokB

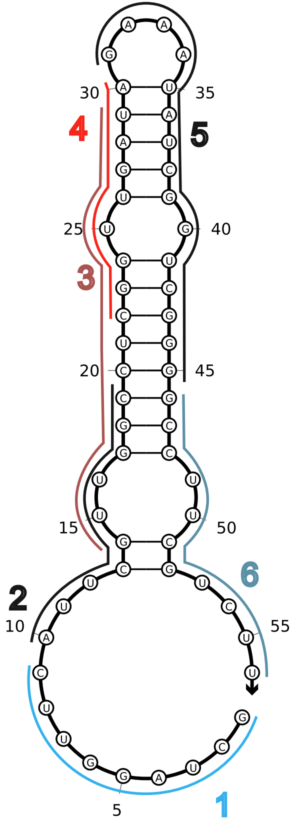

sokC

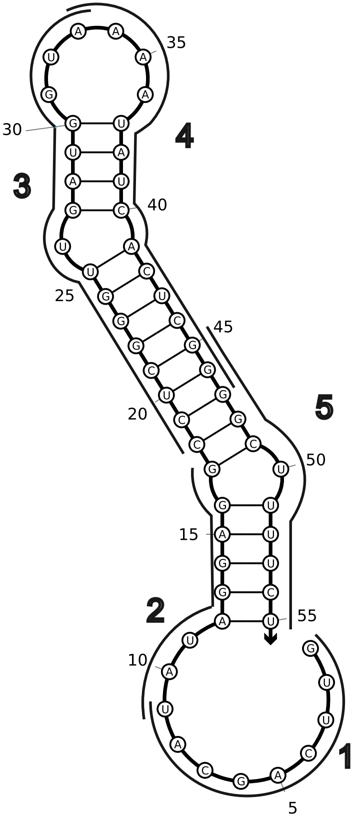

Spot 42

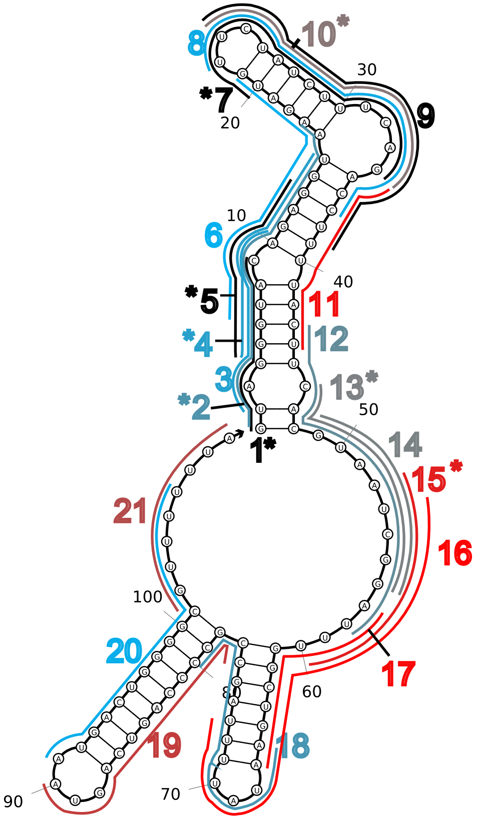

*7
8
10*
9
6
*5
*4
3
*2
1*
11
12
13*
14
15*
16
17
18
19
20
21
10
20
30
40
50
60
70
80
90
100

SraA

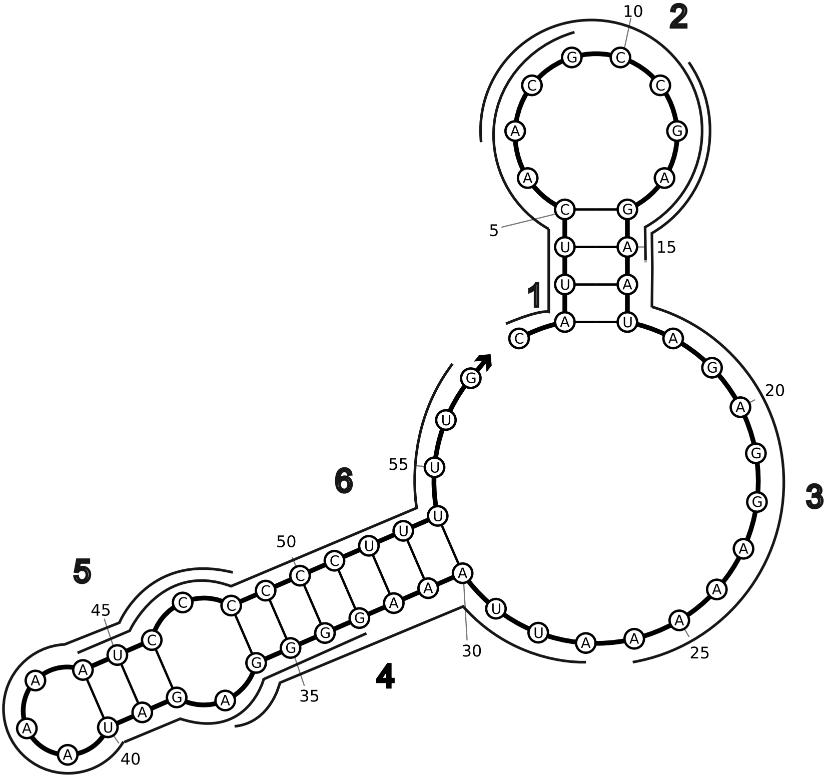

SraD (MicA)

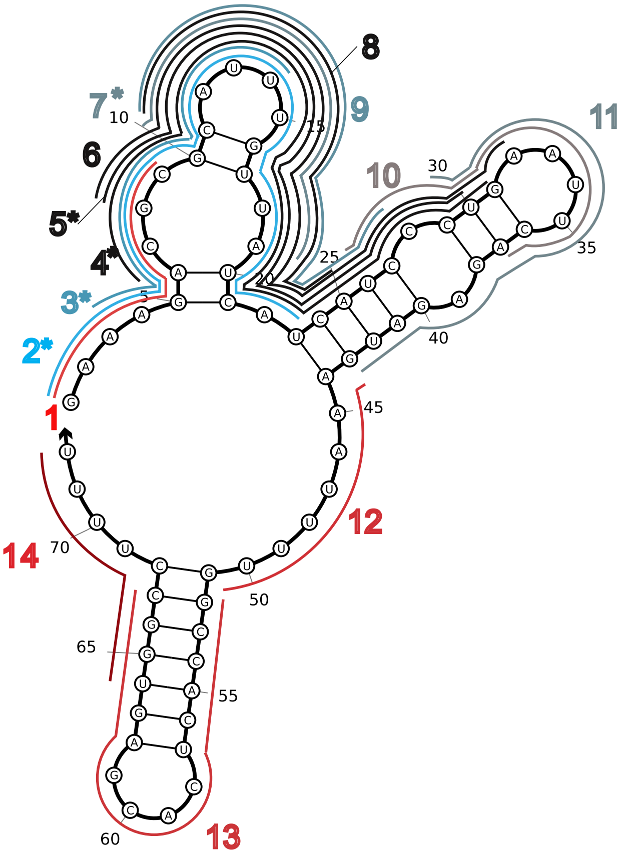

SraH (ArcZ)

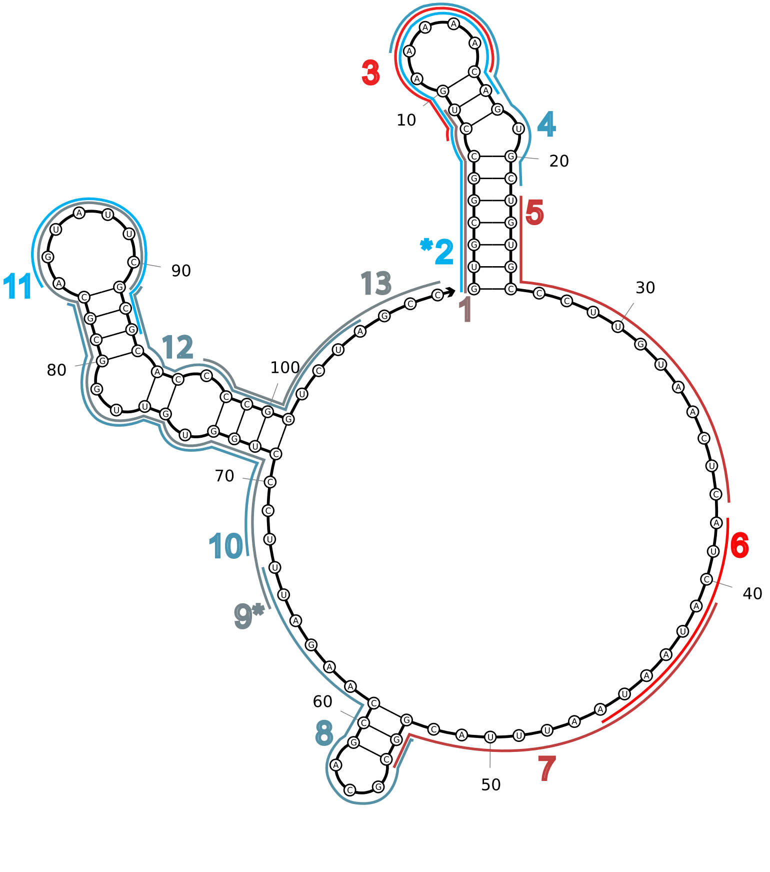

SraL (RyjA)

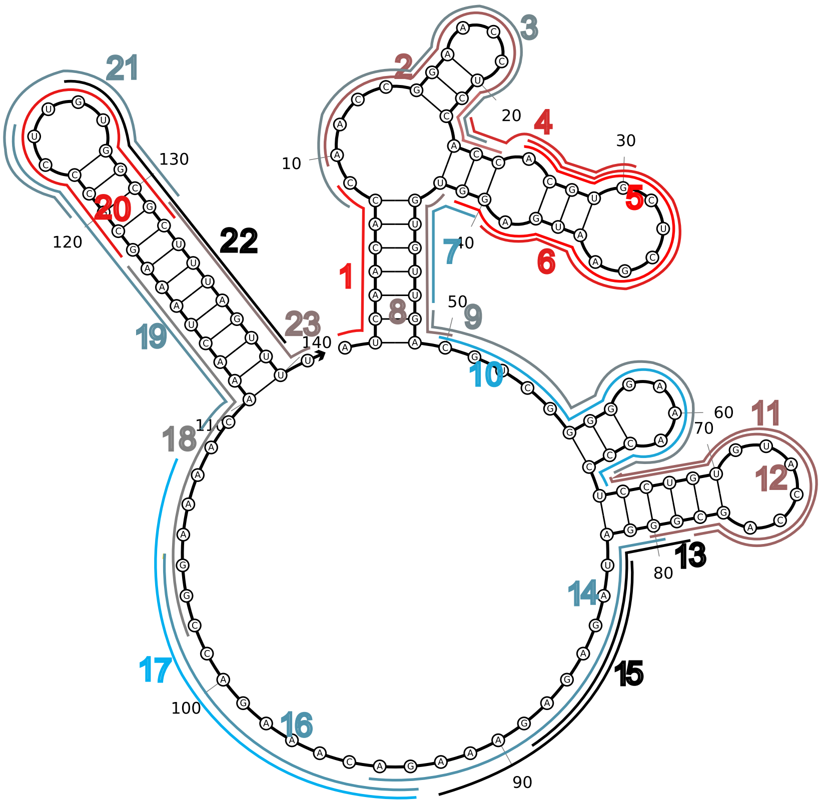

1
2
3
4
5
6
7
8
9
10
11
12
13
14
15
16
17
18
19
20
21
22
23
10
20
30
40
50
60
70
80
90
100
120
130
140

SroA

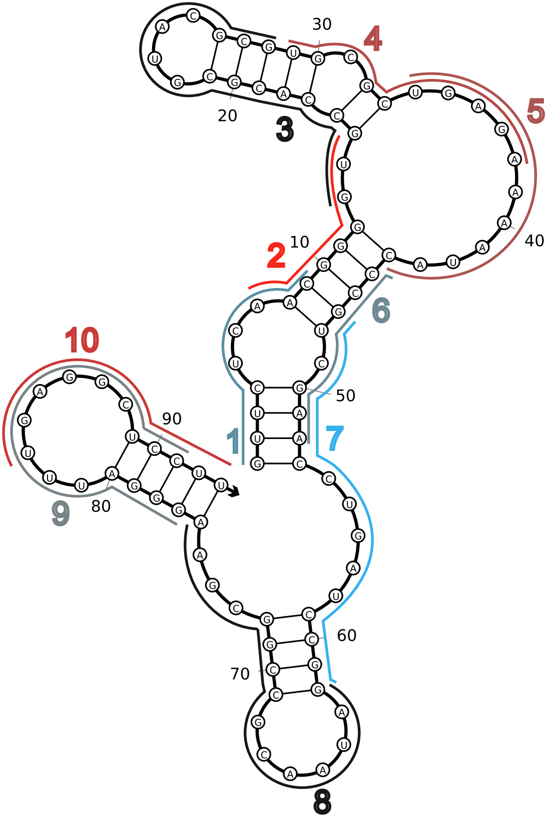

SroB (ChiX)

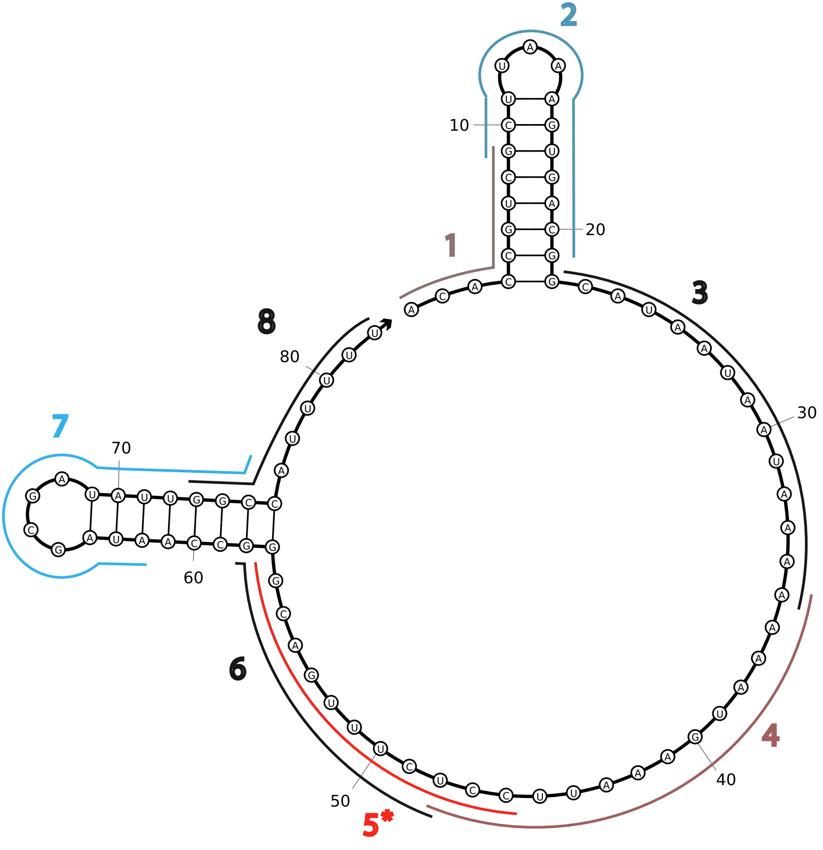

SroC

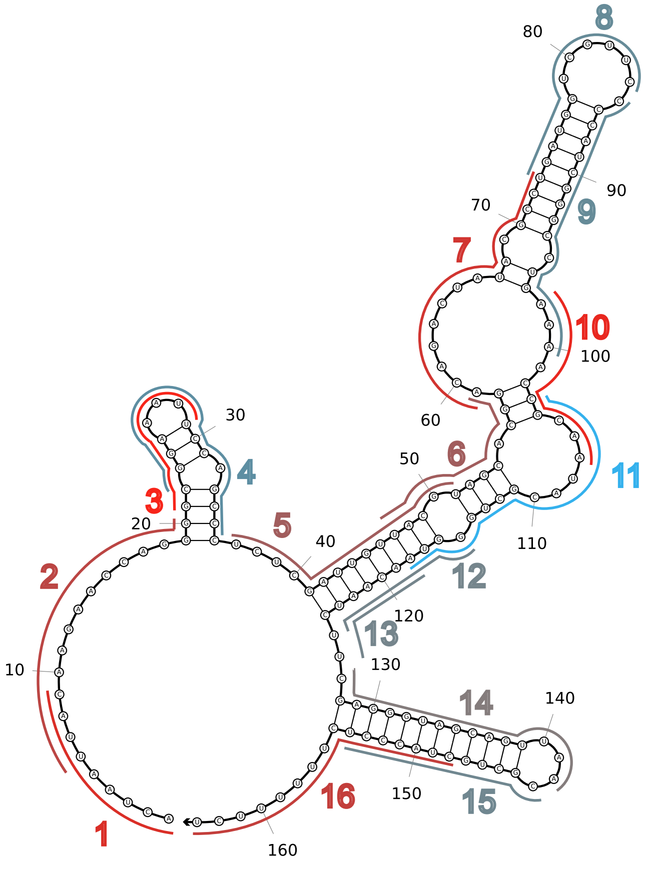

1
2
3
4
5
6
7
8
9
10
11
12
13
14
15
16
10
20
30
40
50
60
70
80
90
100
110
120
130
140
150
160

SroD

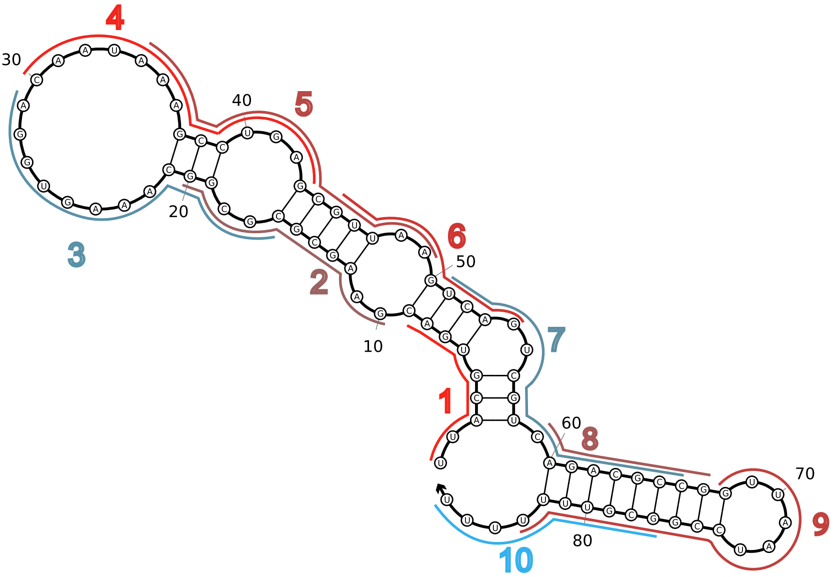

SroE

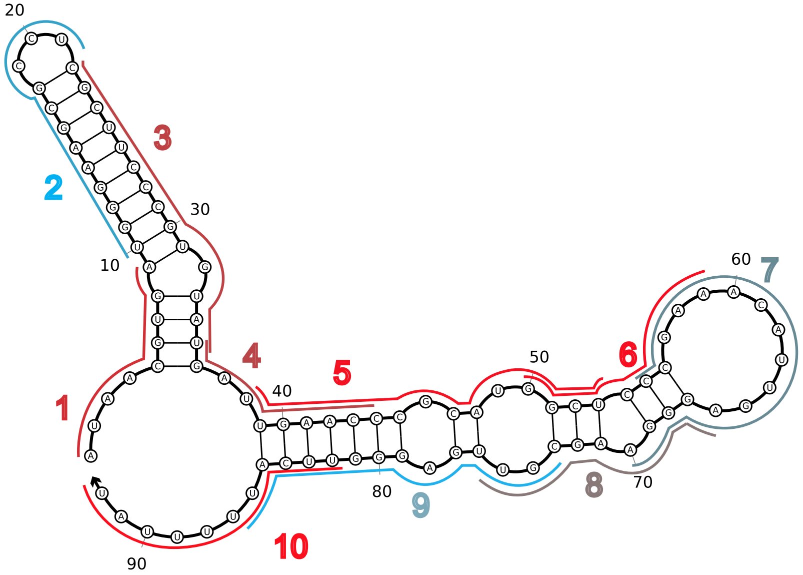

SroG

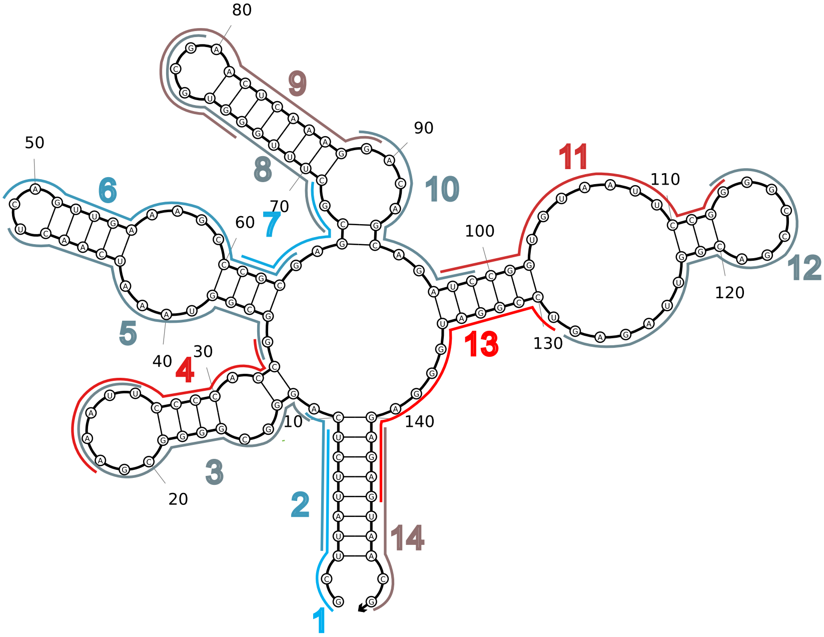

80
9
90
50
6
8
10
11
110
70
7
60
100
12
120
5
13
130
40
30
4
10
140
3
20
2
14
1

SroH

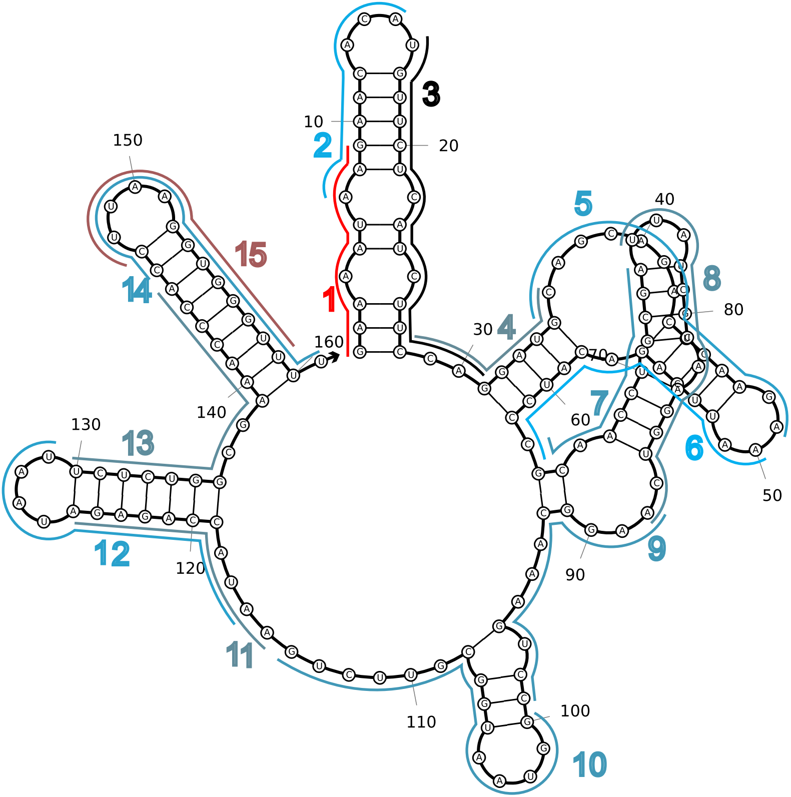

10
20
30
40
50
60
70
80
90
100
110
120
130
140
150
160
1
2
3
4
5
6
7
8
9
10
11
12
13
14
15

SymR

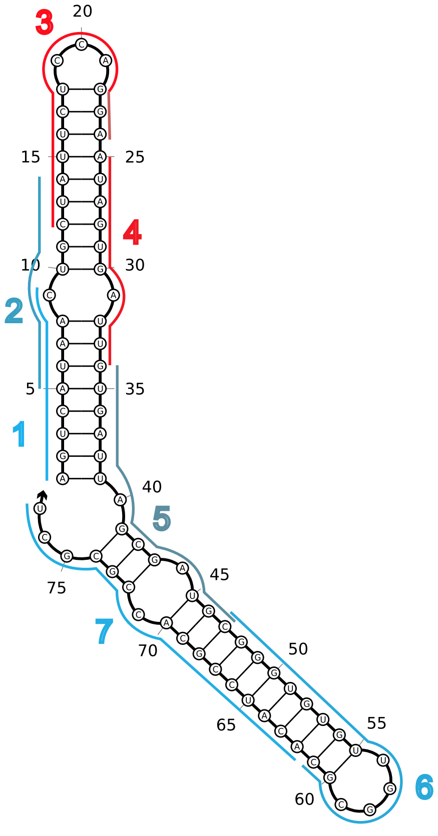

Tff

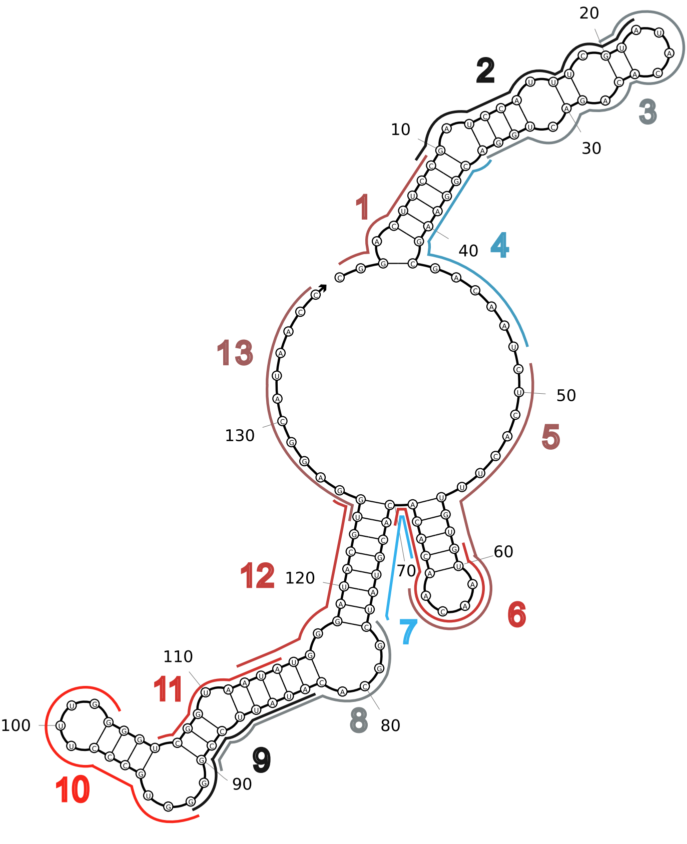

Tp2

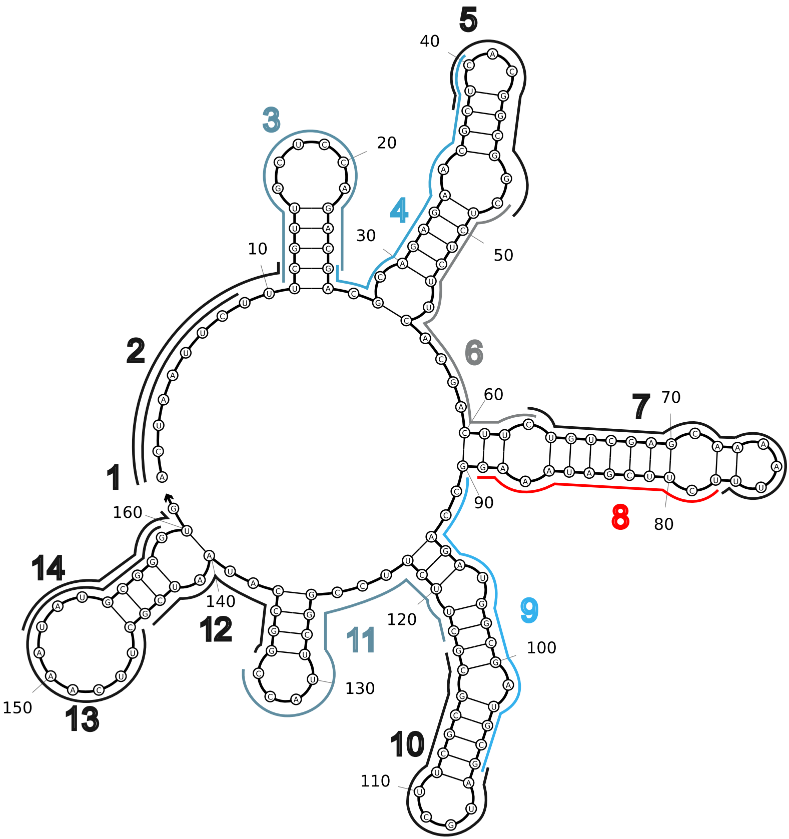

1
2
3
4
5
6
7
8
9
10
11
12
13
14
10
20
30
40
50
60
70
80
90
100
110
120
130
140
150
160

Tpke11

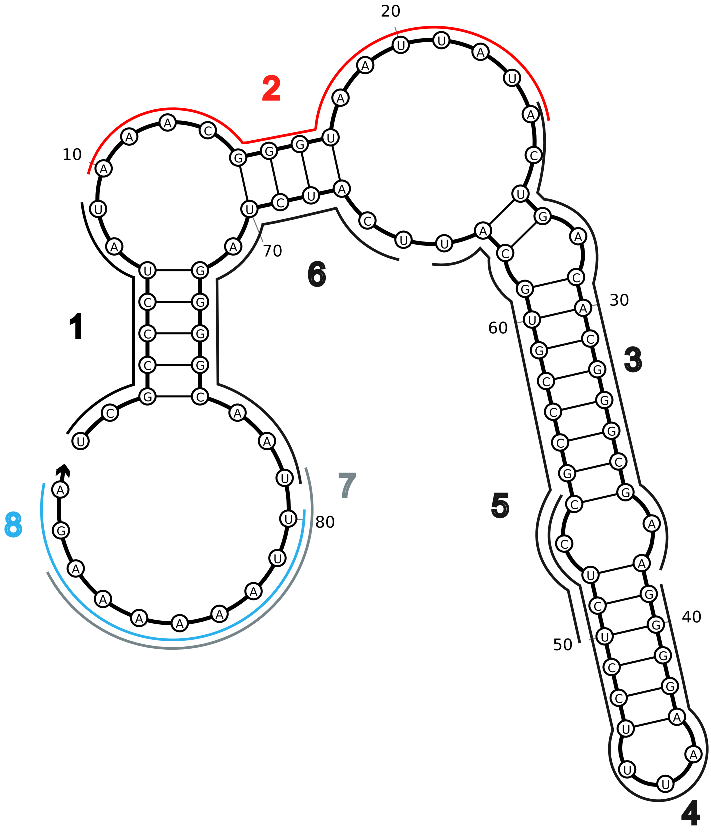

Tpke70

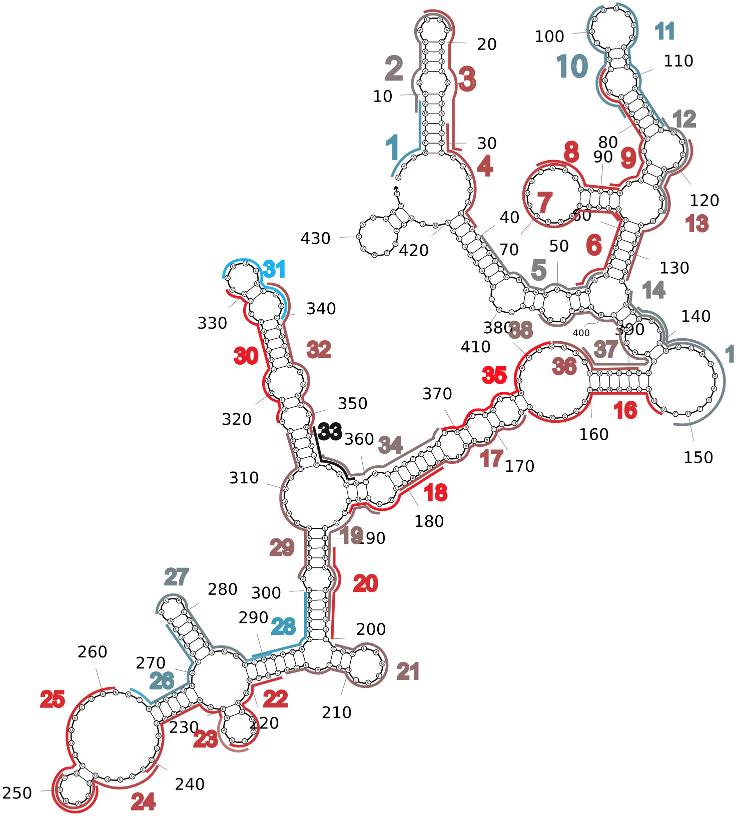

1
2
3
4
5
6
7
8
9
10
11
12
13
14
15
16
17
18
19
20
21
22
23
24
25
26
27
28
29
30
31
32
33
34
35
36
37
38
10
20
30
40
50
70
80
90
100
110
120
130
140
150
160
170
180
190
200
210
220
230
240
250
260
270
280
290
300
310
320
330
340
350
360
370
380
390
400
410
420
430

Supplementary Figure 3. INTERFACE accessibility heat maps for each sRNA molecule analyzed. Red, gray, and blue traces along predicted secondary structures[1] represent high, mid and low accessibility, respectively. Mapped binding sites that composed the training set are indicated with asterisks. To initially test the selectivity of using INTERFACE to propose functional regions, we identified regions exhibiting 5' extreme accessibility end of 3 well-characterized sRNAs that were excluded from our training set (IstR, MicL/RyeF, and RydC). Notably, many of the selected regions agree with confirmed mRNA binding sites; for instance, the confirmed *tisB* mRNA binding site within the sRNA IstR[2] and the *lpp* mRNA binding site within the sRNA MicL/RyeF[3] overlap 5' regions identified as highly accessible (IstR taRNA region 1 and RyeF taRNA region 5, respectively). Furthermore, a likely functional site in sRNA RydC (taRNA region 1) is consistent with a site in its *Salmonella* homologue that is confirmed to interact with the *cfa* leader[4], suggesting the equivalent regulation in *E. coli.*

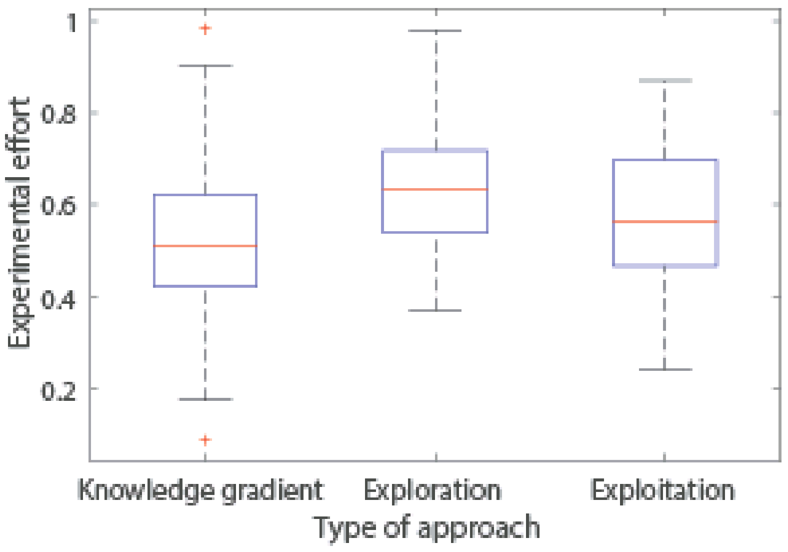

Supplementary Figure 4. Performance of target region design using an SpKG-based algorithm informed by accessibility predictions. Center lines, box limits, whiskers, and red "+" markers of box plots represent the median, lower and upper quartiles, 1.5x interquartile range, and outliers of each group, respectively. Simulated comparisons of regional selection by the described machine learning algorithm to other two other region selection approaches— (i) "relative and random design" (machine learning exploration) or (ii) the adapted biophysical model only (exploitation)— shows that the SpKG approach coupled to a previously-established biophysical model reduced experimental INTERFACE profiling efforts by enriching the number of accessible regions assayed (p-value<0.001, 2-tailed t-test). Furthermore, using exploitation (only the biophysical model) requires less experimental effort than the exploration approach (p-value<0.001, 2-tailed t-test).

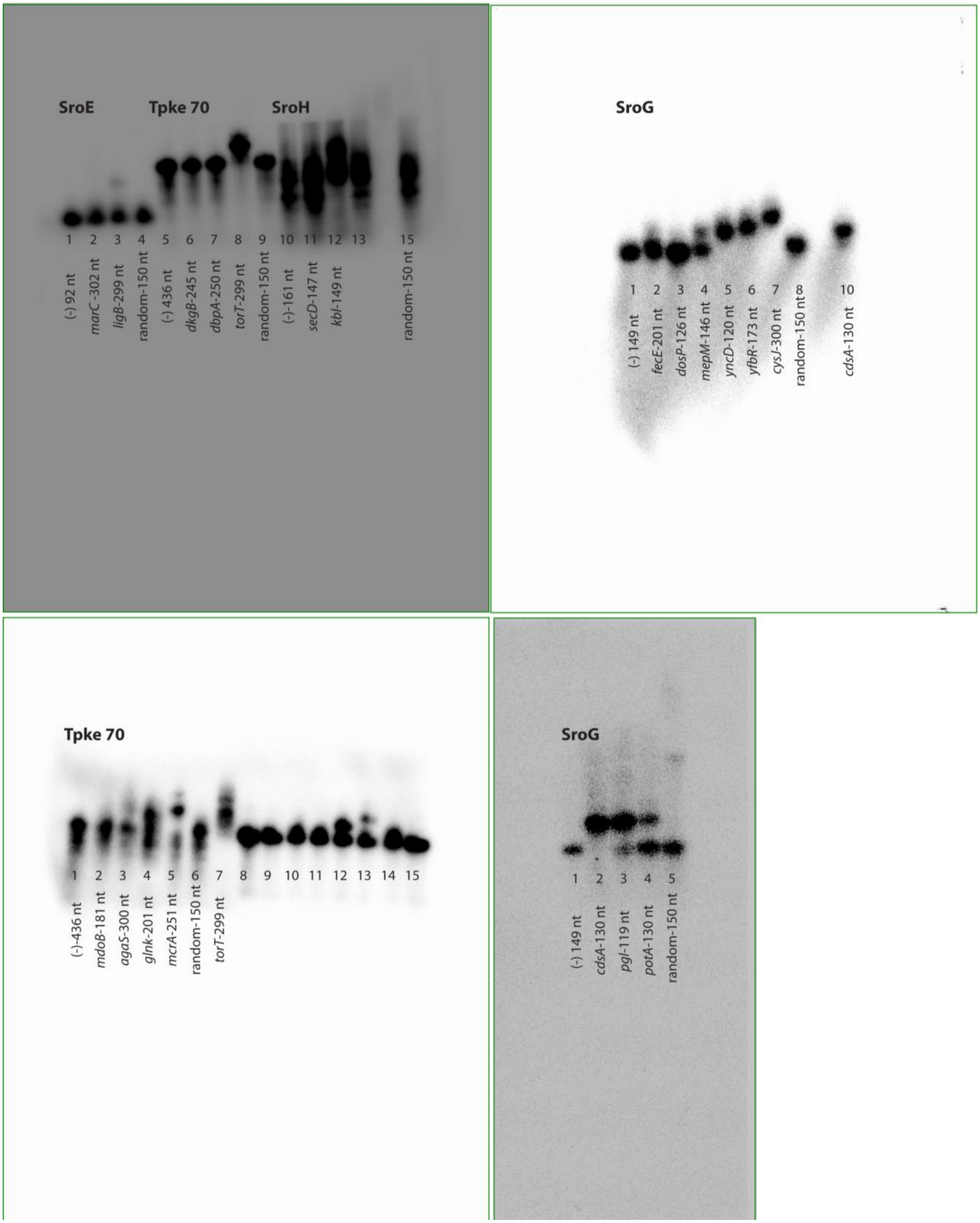
SroE
Tpke 70
SroH
1 2 3 4 5 6 7 8 9 10 11 12 13 15
(-) 92 nt
marC -302 nt
ligB-299 nt
random-150 nt
(-) 436 nt
dkgB-245 nt
dbpA-250 nt
torT-299 nt
random-150 nt
(-)-161 nt
secD-147 nt
kbl-149 nt
random-150 nt
SroG
1 2 3 4 5 6 7 8 10
(-) 149 nt
fecE-201 nt
dosP-126 nt
mepM-146 nt
yncD-120 nt
yfbR-173 nt
cysJ-300 nt
random-150 nt
cdsA-130 nt
Tpke 70
1 2 3 4 5 6 7 8 9 10 11 12 13 14 15
(-)-436 nt
mdoB-181 nt
agaS-300 nt
glnk-201 nt
mcrA-251 nt
random-150 nt
torT-299 nt
SroG
1 2 3 4 5
(-) 149 nt
cdsA-130 nt
pgl-119 nt
potA-130 nt
random-150 nt

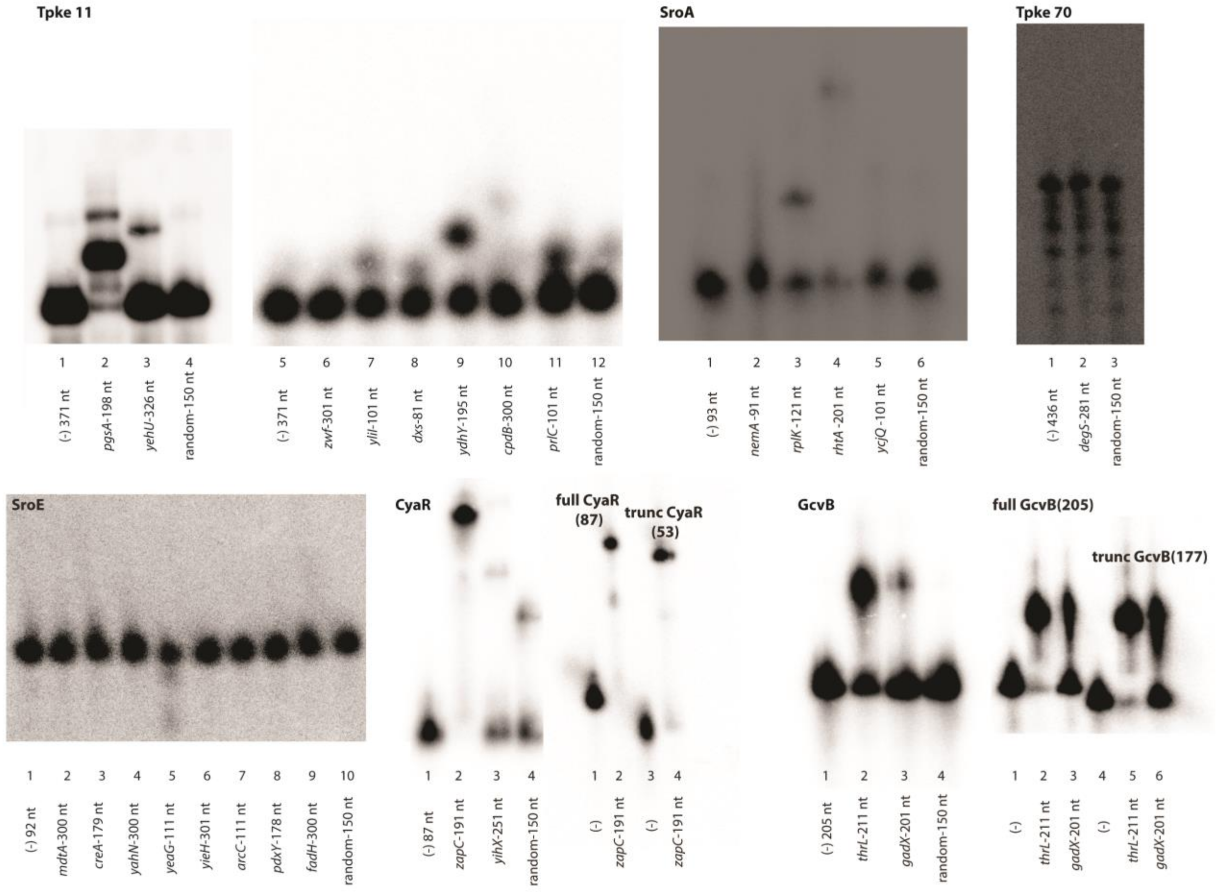

Supplementary Figure 5. Consecutive lanes of all EMSAs performed for this study. Sequential lanes of all EMSAs (corresponding to unique sRNAs) referenced in the manuscript are pictured, excluding those for GlmY (Fig. 6 in manuscript). Full-gel images are shown for the three benchmarked under-characterized sRNAs (top). Unlabeled lanes 8-15 in bottom left full-gel image were rerun for greater spatial resolution (Tpke11 lanes 5-12, equivalent order) due to the large size of Tpke11 coupled with the small sizes of some corresponding predicted targets. Length of all sRNAs are listed below negative sRNA-only control lanes (indicated by "(-)" below the corresponding well), with the exception of gels containing truncated sRNAs, in which case the length of the sRNA is indicated above corresponding wells. Identity and length of all mRNAs tested are indicated underneath respective gel lanes.

Supplementary References

1. Zadeh, J.N. et al. NUPACK: Analysis and design of nucleic acid systems. *Journal of Computational Chemistry* **32**, 170-173 (2011).
2. Darfeuille, F., Unoson, C., Vogel, J. & Wagner, E.G.H. An Antisense RNA Inhibits Translation by Competing with Standby Ribosomes. *Molecular Cell* **26**, 381-392 (2007).
3. Guo, M.S. et al. MicL, a new σ(E)-dependent sRNA, combats envelope stress by repressing synthesis of Lpp, the major outer membrane lipoprotein. *Genes & Development* **28**, 1620-1634 (2014).
4. Fröhlich, K.S., Papenfort, K., Fekete, A. & Vogel, J. A small RNA activates CFA synthase by isoform-specific mRNA stabilization. *The EMBO Journal* **32**, 2963-2979 (2013).
