# Supplementary material for: High-throughput in vivo mapping of RNA accessible interfaces to identify functional sRNA binding sites
Source: Nat Commun. 2018 Oct 4;9:4084. doi: 10.1038/s41467-018-06207-z (PMC6172242; doi:10.1038/s41467-018-06207-z)
Supplement: Supplementary file 3 — Description of Additional Supplementary Files [file 41467_2018_6207_MOESM3_ESM.pdf]

Description of Additional Supplementary Files

File Name: Supplementary Data 1
Description: **List of sRNAs in this study and relevant information on sRNA:mRNA binding sites and Hfq-dependence.** Name and sequence for all sRNAs probed in this study are listed. Reported Hfq dependency and corresponding footprinted and/or predicted Hfq binding sites from E. coli or Salmonella studies are annotated within the sRNA sequence with bold or underline (corresponding to first and second validation/prediction source, respectively). For each sRNA, confirmed targets in E. coli and corresponding nucleotides of interaction, functional effect, and validation method are listed. All binding partners and positions not obtained from sRNATarBase5 are referenced. Sixteen sRNAs and corresponding intervals of mapped binding sites that were used as a training set are bolded.

File Name: Supplementary Data 2
Description: **Accessibility values for all sRNA regions probed with INTERFACE.** Reported position, normalized accessibility values, replicate number, and standard deviation for all targeted sRNA regions, in both BW25113 and isogenic BW25113 Hfq-knockout strain

File Name: Supplementary Data 3
Description: **Summary of IntaRNA-predicted target mRNAs chosen for experimental validation via EMSA.** All relevant parameters of experimentally tested IntaRNA predictions are listed, including prediction rank and predicted energy, sRNA details (sRNA name, sequence, predicted binding interval), and mRNA details (annotated mRNA name, gene name, mRNA sequence used in IntaRNA prediction and the adjusted mRNA sequence chosen for use in assays based on previously obtained transcript expression profiles, and the mRNA nucleotides predicted to interact). Predicted binding intervals are highlighted in red within sRNA and mRNA sequences, and mRNA start codons are highlighted in green. Truncated sRNA sequences used as sRNA TER-only sequence inputs for CyaR, GcvB, and GlmY predictions are listed.

File Name: Supplementary Data 4
Description: **Comparison of Hfq dependencies between literature and INTERFACE experiments.** Literature-based Hfq-dependencies, in which strong, mid, no, or inconsistent Hfq dependence (based on experimental techniques separated by column) is highlighted green, orange, red, and yellow, respectively, are compared to INTERFACE-based dependencies for the ensemble of sRNAs probed in this work. Other mechanistic characteristics related to Hfq dependency (ie., cis v. trans regulation, known dependence on other common RNA-binding proteins, known Hfq dependency class as based on interaction behavior) are also listed for each sRNA. sRNAs SokC and SraA are not included due to insufficient data from the INTERFACE experiment, likely due to limited sequencing depth.

File Name: Supplementary Data 5
Description: **Annotated plasmid map of O-INTERFACE.**

File Name: Supplementary Data 6
Description: **Annotated plasmid map of INTERFACE.**

File Name: Supplementary Data 7
Description: **Primers used in this study.** Primers used for the insertion of asRNAs to OINTERFACE (taRNA = gI intron) or INTERFACE (taRNA = sRNAs) plasmids via Golden Gate cloning are compiled. Primers for the amplification of sRNAs/mRNAs (or gBlock (IDT) sequences) for use in IVT are separated into two groups (undercharacterized sRNAs v. well-characterized).
